# Potassium sodium hydrogen citrate intervention on gut microbiota and clinical features in uric acid stone patients

**DOI:** 10.1007/s00253-023-12953-y

**Published:** 2024-01-06

**Authors:** Cheng Cao, Feng Li, Qi Ding, Xiaohua Jin, Wenjian Tu, Hailiang Zhu, Mubin Sun, Jin Zhu, Dongrong Yang, Bo Fan

**Affiliations:** 1 Department of Urology, The Changshu Hospital Affiliated to Soochow University (Changshu No. 1 People’s Hospital), Changshu, China; 2 Department of Urology, The Second Affiliated Hospital of Soochow University, Suzhou, China

**Keywords:** Gut microbiota, Renal uric acid stone, 16S rRNA, Short chain fatty acid, Potassium sodium hydrogen citrate

## Abstract

**Abstract:**

The high recurrence rate of renal uric acid stone (UAS) poses a significant challenge for urologists, and potassium sodium hydrogen citrate (PSHC) has been proven to be an effective oral dissolution drug. However, no studies have investigated the impact of PSHC on gut microbiota and its metabolites during stone dissolution therapy. We prospectively recruited 37 UAS patients and 40 healthy subjects, of which 12 patients completed a 3-month pharmacological intervention. Fasting vein blood was extracted and mid-stream urine was retained for biochemical testing. Fecal samples were collected for 16S ribosomal RNA (rRNA) gene sequencing and short chain fatty acids (SCFAs) content determination. UAS patients exhibited comorbidities such as obesity, hypertension, gout, and dyslipidemia. The richness and diversity of the gut microbiota were significantly decreased in UAS patients, *Bacteroides* and *Fusobacterium* were dominant genera while *Subdoligranulum* and *Bifidobacterium* were poorly enriched. After PSHC intervention, there was a significant reduction in stone size accompanied by decreased serum uric acid and increased urinary pH levels. The abundance of pathogenic bacterium *Fusobacterium* was significantly downregulated following the intervention, whereas there was an upregulation observed in SCFA-producing bacteria *Lachnoclostridium* and *Parasutterella*, leading to a significant elevation in butyric acid content. Functions related to fatty acid synthesis and amino acid metabolism within the microbiota showed upregulation following PSHC intervention. The correlation analysis revealed a positive association between stone pathogenic bacteria abundance and clinical factors for stone formation, while a negative correlation with SCFAs contents. Our preliminary study revealed that alterations in gut microbiota and metabolites were the crucial physiological adaptation to PSHC intervention. Targeted regulation of microbiota and SCFA holds promise for enhancing drug therapy efficacy and preventing stone recurrence.

**Key points:**

*• Bacteroides and Fusobacterium were identified as dominant genera for UAS patients*

*• After PSHC intervention, Fusobacterium decreased and butyric acid content increased*

*• The microbiota increased capacity for fatty acid synthesis after PSHC intervention*

**Supplementary Information:**

The online version contains supplementary material available at 10.1007/s00253-023-12953-y.

## Introduction

Urolithiasis is a globally prevalent disease, with reported prevalence rates ranging from 7 to 13% in North America, 5 to 9% in Europe, and 1 to 5% in Asia based on epidemiological studies (Abufaraj et al. 2022; Sorokin et al. 2017). However, a consistent upward trend in the overall incidence has been observed regions (Scales et al. 2012; Yasui et al. 2008). Notably, the southern region of China has exhibited an alarming prevalence exceeding 10% (Ye et al. 2020). The composition of nephrolithiasis primarily consists of calcium-containing stones, with calcium oxalate stones being predominant and accounting for over 80%. Uric acid stones (UAS) account for 5 to 15% of all kidney stones (Ma et al. 2018a). In the USA, the percentage of UAS has remained at approximately 10%, while in China, they account for about 5.1% of all stone components; however, this proportion increases to more than 10% in the southern region (Ma et al. 2018b). These epidemiological differences suggest that geography, climate, race, and other factors may be linked to UAS formation.

Uric acid is the end product of purine degradation. Humans, unlike other mammals, lack the activity of uric acid oxidase, resulting in an inability to convert uric acid into its more soluble form allantoin and leading to the formation of UAS (Ramos and Goldfarb et al. 2022). The formation process is influenced by urine volume levels, pH levels, and uric acid concentrations. Persistent over-acidification plays a decisive role in this regard. Epidemiologic studies have shown that metabolic syndrome is strongly associated with UAS due to various metabolic and environmental factors promoting insulin resistance and stone formation within urine samples (Abou-Elela 2017; Bamberger et al. 2021; Xiao et al. 2020). Stone patients who are obese or diabetic tend to have higher proportions of total stones consisting of UAS compared to those who possess normal body mass indices (BMI) or serum glucose levels (Daudon et al. 2006a, 2006b). The gut microbiota not only plays a pivotal role in digestion but also exerts a profound influence on the pathogenesis of metabolic disorders such as hypertension, diabetes mellitus, hyperlipidemia, and urolithiasis (Chen et al. 2021b; Hong et al. 2023). It has been observed that stone patients exhibit alterations in both diversity and species composition of their gut microbiota, characterized by significant reductions in flora involved in oxalic acid degradation and SCFA (short chain fatty acid) production (Chen et al. 2021a; Miller et al. 2019).

UAS is the only type of stone with definitive evidence supporting treatment with oral alkaline medications for dissolution. Potassium sodium hydrogen citrate (PSHC), the most representative alkaline citrate preparation, is recommended as the preferred therapeutic agent for UAS patients (Turk et al. 2016), with oral dissolution rates ranging from 73 to 100% (Nevo et al. 2020). Alkalizing urine can effectively promote UAS dissolution by increasing uric acid solubility approximately 20-fold as urine pH increases from 5.0 to 7.0. Furthermore, PSHC remains a feasible option for residual stones after surgery and X-ray negative stones in cases where surgery is contraindicated (Elsawy et al. 2019).

Although extensive research has been conducted on the characteristics of gut microbiota and metabolites in patients with calcium oxalate stones, limited studies have explored these factors in individuals with UAS who undergo oral dissolution therapy with PSHC. Therefore, we conducted a preliminary study to analyze the gut microecological and biochemical features of UAS patients before and after PSHC intervention in order to investigate changes in gut microbiota, metabolites, and biochemical parameters.

## Materials and methods

### Subjects and sample collection

This study was approved by the Ethics Review Committee of Changshu Hospital affiliated with Soochow University. A prospective screening was conducted from September 2019 to August 2022, including UAS patients and a healthy population as participants who provided informed consent. All stone patients were diagnosed using urologic ultrasonography, kidney-ureter-bladder (KUB) X-ray, or abdominal computed tomography (CT). Stone samples were obtained through ureteroscopic lithotripsy (URSL), percutaneous nephrolithotomy (PCNL), or extracorporeal shock wave lithotripsy (ESWL). Stones were analyzed using an automated infrared spectroscopy system, LIIR-20 (Lanmode Scientific Instrument Co., Ltd., Tianjin, China), and the main components were determined based on the most abundant substances listed in the report, which were classified as pure or mixed uric acid stones (anhydrous uric acid content > 50%). Patients with malignancy, chronic liver insufficiency, a history of statin use, and thyroid or parathyroid disease were excluded from the study. Similarly, individuals with a history of urolithiasis or dyslipidemia, as well as those who had used statins, were excluded from the control group. Participants were also excluded if they had taken antibiotics or immune suppressants within one month prior to fecal sampling, or had a history of chronic diarrhea or constipation, chronic enteritis, irritable bowel syndrome, gastrointestinal tumors, or intestinal surgery (Supplemental Fig. S1A).

PSHC granules (MADAUS GMBH, Cologne, Germany, 2.5 g/packet) were administered to patients with residual stones (> 5 mm in diameter) after surgery or difficult-to-remove stones in the infrarenal calyces for a 3-month intervention period. The medication was taken as follows: one packet after breakfast, one packet after lunch, and two packets after dinner. Participants were instructed to abstain from alcohol consumption, smoking, and the use of any probiotics or medications that lower uric acid levels during the therapeutic period. They were also instructed to monitor preprandial urine pH levels to maintain an effective range of 6.2–6.8 (Kamphuis et al. 2019).

A total of 37 UAS patients were prospectively recruited (UAS group), and 40 non-stone subjects who underwent physical examination at Changshu Hospital affiliated with Soochow University were enrolled via age and gender matching (NS group). Among the 37 UAS patients, 17 individuals diagnosed as pure anhydrous uric acid stone formers fulfilled the criteria for treatment with PSHC.

General characteristics, including height, weight, and past medical history, were collected from all participants. After consuming a standard diet provided for 3 days (Supplemental Table S1), fasting venous blood (approximately 5 ml) was drawn from each participant and mid-stream urine was retained for follow-up testing. Meanwhile, fresh fecal samples (5 g) were collected from the subjects and stored at − 80 °C in tubes preloaded with fecal DNA stabilizer until analysis.

### Fecal 16S rRNA sequencing and SCFAs determination

The DNA was extracted from 200 mg of fecal samples using the QIAamp DNA kit (QIAGEN, Hilden, Germany). Subsequently, the extracted DNA was analyzed by 1.2% agarose gel electrophoresis to confirm its quality and integrity. For library construction, a two-step PCR amplification method targeting the V3-V4 region of the 16S rRNA gene was employed. The PCR amplification utilized universal primers 357F (5′-ACTCCTACGGRAGGCAGCAG-3′) and 806R (5′-GGACTACHVGGGTW TCTAAT-3′). All resulting PCR products were subsequently purified using the AxyPrepDNA Gel Recovery Kit (AXYGEN, San Francisco, USA). Fluorescence quantification was performed using the FTC-3000TM Real-Time PCR instrument (Funglyn, Shanghai, China), followed by PCR amplification and library construction. The constructed libraries were sequenced on a Novaseq 6000 SP 500 Cycle Reagent Kit (Illumina, San Diego, USA) platform following standard protocols established by TinyGen Bio-Tech (Shanghai, China) Co., Ltd. Fecal SCFAs contents were quantified via gas chromatography-mass spectrometry (GC–MS) analysis utilizing the Thermo Trace 1300 gas chromatograph and Thermo ISQ 7000 mass spectrometer (Thermo Fisher Scientific, Wilmington, USA). The components separated by chromatography were continuously introduced into the mass spectrometer, and a continuous scan of the mass spectrometer was conducted for data acquisition. A calibration curve was constructed using the concentration of the standard as the x-coordinate and the peak area ratio of the standard to the internal standard as the y-coordinate. Based on these established metabolite calibration curves, quantitative calculations were performed for all samples to determine the contents of SCFAs in each fecal sample (*R*^2^ > 0.99).

### Statistical and bioinformatic analysis

The general characteristics and biochemical parameters of the subjects were analyzed using the SPSS 22.0 software (IBM, Armonk, NY, USA). Statistical significance was determined at a two-sided *P* < 0.05 level. Data normality was assessed using the Shapiro–Wilk test, and continuous variables that followed a normal distribution were presented as mean ± SD, while non-normally distributed variables were presented as median and interquartile range (IQR). Comparisons between continuous variables were conducted using either Student’s *t*-test or Mann–Whitney test, depending on data distribution. Categorical variables were evaluated using either chi-square test or Fisher’s exact test.

The 16S rRNA sequences were analyzed using mothur software (University of Michigan, Ann Arbor, USA, version 1.33.3) in conjunction with R language (version 3.6.3; https://cran.r-project.org/bin/windows/base/old/). The sequences were clustered into operational taxonomic units (OTUs) at a 97% similarity threshold using the UPARSE software (University of Lyon, Lyon, France, usearch version 8.1.1756; http://drive5.com/uparse/) and R language (version 3.6.3). Taxonomic analysis of community species was performed at the kingdom, phylum, class, order, family, genus, and species levels based on the National Center for Biotechnology Information (NCBI, Bethesda, Maryland, USA; https://www.ncbi.nlm.nih.gov/) database and taxonomic information. Alpha diversity indices, including ACE, Chao, Shannon, and Simpson were computed using mothur software (version 1.33.3) and R language (version 2.5–6; https://cran.r-project.org/web/packages/vegan/vegan.pdf). The UniFrac distance matrix was generated through Partial Least Squares Discriminant Analysis (PLS-DA) in R language (version 1.18.0; https://bioconductor.org/packages/release/ bioc/html/ropls.html) to reflect the β-diversity of community differences between samples. Linear discriminant analysis Effect Size (LEfSe) was employed to identify significantly enriched microbiota in each group (Harvard University, Cambridge, MA, USA; https://huttenhower.sph.harvard.edu/lefse). In LEfSe analysis, linear discriminant analysis (LDA) was conducted with a cutoff value defined as 3.0 to screen for genera that had significant effects on inter-group differences. The functional prediction of gut microbiota based on 16S rRNA sequencing was conducted using the Phylogenetic Investigation of Communities by Reconstruction of Unobserved States (PICRUSt, Harvard University, Cambridge, MA, USA; https:// huttenhower.sph.harvard.edu/picrust/) algorithm in the Kyoto Encyclopedia of Genes and Genomes (KEGG, Kyoto University, Kyoto City, Japan; https://www.genome.jp/ kegg/) database. Spearman correlations between differentially abundant genera and biochemical features, as well as SCFA contents, were analyzed (psych package, version 1.8.12; https://www.rdocumentation.org/packages/psych/ versions/1.8.12) and visualized through heatmaps (pheatmap package, version 1.0.12; https://www.rdocumentation.org/packages/pheatmap/versions/1.0.12/topics/pheatmap).

## Results

### General characteristics and biochemical features of the study population

The prevalence of BMI (*P* < 0.001), hypertension (*P* = 0.044), and gout (*P* < 0.001) was significantly higher in patients with UAS compared to healthy controls. In terms of biochemical features, UAS patients exhibited elevated levels of serum triglycerides (TG) (*P* < 0.001), serum potassium (*P* = 0.044), serum creatinine, and serum uric acid (both *P* < 0.001). Additionally, they had lower levels of serum high-density lipoprotein cholesterol (HDL-C) (*P* < 0.001). Typical acidic urine was observed in UAS patients along with a significant increase in urinary leukocyte count (*P* < 0.001) (Table 1).

**Table 1:** Comparison of general characteristics and biochemical features between UAS patients and NS controls

Variables	UAS patients	NS controls	*P* value
	(n = 37)	(*n* = 40)	
Age (years, mean ± SD)	53.89 ± 13.93	49.08 ± 12.70	0.117
BMI (kg/m^2^, mean ± SD)	25.98 ± 2.90	23.30 ± 2.37	**< 0.001**
Gender (%)			0.576
Male	28 (75.68)	28 (70.00)	
Female	9 (24.32)	12 (30.00)	
Hypertension (%)			**0.044**
Yes	16 (43.24)	9 (22.50)	
No	21 (56.76)	31 (77.50)	
Diabetes (%)			0.419
Yes	4 (10.81)	2 (5.00)	
No	33 (89.19)	38 (95.00)	
Gout (%)			**< 0.001**
Yes	21 (56.76)	3 (7.50)	
No	16 (43.24)	37 (92.50)	
Serum lipid levels, mmol/L			
TG	1.90 (1.36)	1.27 (1.00)	**< 0.001**
TC	4.95 ± 1.19	4.94 ± 0.82	0.967
HDL-C	1.16 ± 0.28	1.47 ± 0.36	< 0.001
LDL-C	2.91 ± 0.80	2.91 ± 0.74	0.965
Serum electrolyte levels, mmol/L			
K	4.31 ± 0.61	4.09 (0.25)	**0.044**
Na	140.77 ± 2.57	140.16 ± 1.68	0.228
Cl	104.29 ± 3.30	103.60 ± 2.18	0.284
Ca	2.36 (0.13)	2.31 (0.21)	0.143
P	1.09 (0.31)	1.10 (0.13)	0.535
Mg	0.87 (0.09)	0.92 (0.06)	0.119
Serum Cr, μmol/L	98.57 ± 36.16	73.38 ± 16.95	**< 0.001**
Serum UA, μmol/L	512.84 ± 104.54	365.75 ± 91.69	**< 0.001**
Urinary pH	5.00 (1.00)	6.00 (0.50)	**< 0.001**
Urinary WBC count, /μL	19.00 (82.00)	2.00 (3.50)	**< 0.001**

Statistical comparisons were performed using Student’s *t*-test, Mann–Whitney *U* test, and two-sided chi-square test

*UAS*, uric acid stone; *NS*, non-kidney stone; *BMI*, body mass index; *TG*, triglycerides; *TC*, total cholesterol; *HDL-C*, high-density lipoprotein cholesterol; *LDL-C*, low- density lipoprotein cholesterol; *Cr*, creatinine; *UA*, uric acid; *WBC*, white blood cell

Bold values indicate significant differences

### Analysis of gut microbiota richness and diversity in UAS patients compared to healthy controls

**Fig. 1:**
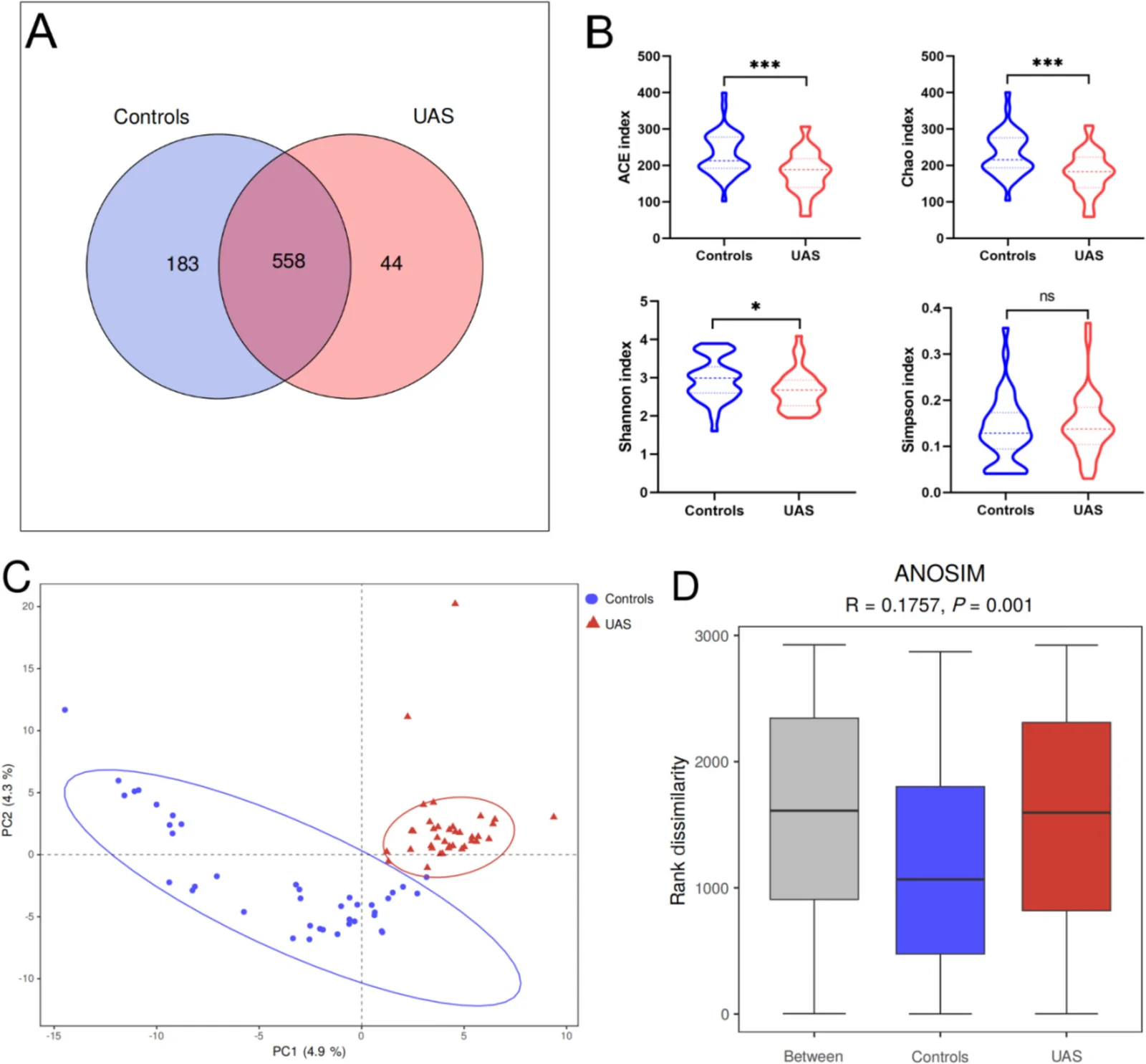
Richness and diversity of gut microbiota between UAS patients and controls by using 16S rRNA. **A** The Venn diagram illustrated the shared and unique OTUs between UAS patients and controls based on 16S rRNA sequencing. **B** The α-diversity indices, including ACE index, Chao index, Shannon index, and Simpson index, were compared between UAS patients and controls using the Kruskal–Wallis test (*P < 0.05, **P < 0.01, ***P < 0.001). **C** PLS-DA analysis revealed distinct clustering of samples from different groups based on their microbial composition variations represented by dots of different colors and shapes in a two-dimensional space defined by PC1 and PC2. **D** The Anosim test confirmed that intergroup differences in species composition were significantly greater than intragroup differences, validating the rationality of the grouping methodology

After eliminating repetitive and unclear sequences that could affect the quality of analysis, a total of 3,843,517 high-quality sequences with an average length of 419 were obtained through 16S rRNA high-throughput sequencing of 77 fecal samples. The sequencing identified a total of 934 OTUs, including one kingdom, 13 phyla, 22 classes, 30 orders, 50 families, 150 genera, and 188 species. The Venn diagram in Fig. 1A illustrated the unique and shared OTUs among different groups. Out of the total 785 known OTUs, the UAS group exhibited a significantly lower number of OTUs compared to the control group, indicating a reduced richness of gut microbiota in patients with UAS.

The α-diversity indices, including ACE, Chao, Shannon, and Simpson, reflected different aspects of microbial diversity in a community. While the ACE and Chao indexes indicated species richness, i.e., the number of species present in the community, the Shannon and Simpson indices were more indicative of species diversity. Our results showed significantly lower gut microbiota richness and diversity in UAS patients compared to controls (Fig. 1B). PLS-DA visualization analysis and Anosim non-parametric test (*R* = 0.176, *P* = 0.001) based on OTU level revealed significant differences in overall microbiota composition between the two groups (Fig. 1C–D).

### Comparative taxonomic analysis of gut microbiota composition between the two groups

**Fig. 2:**
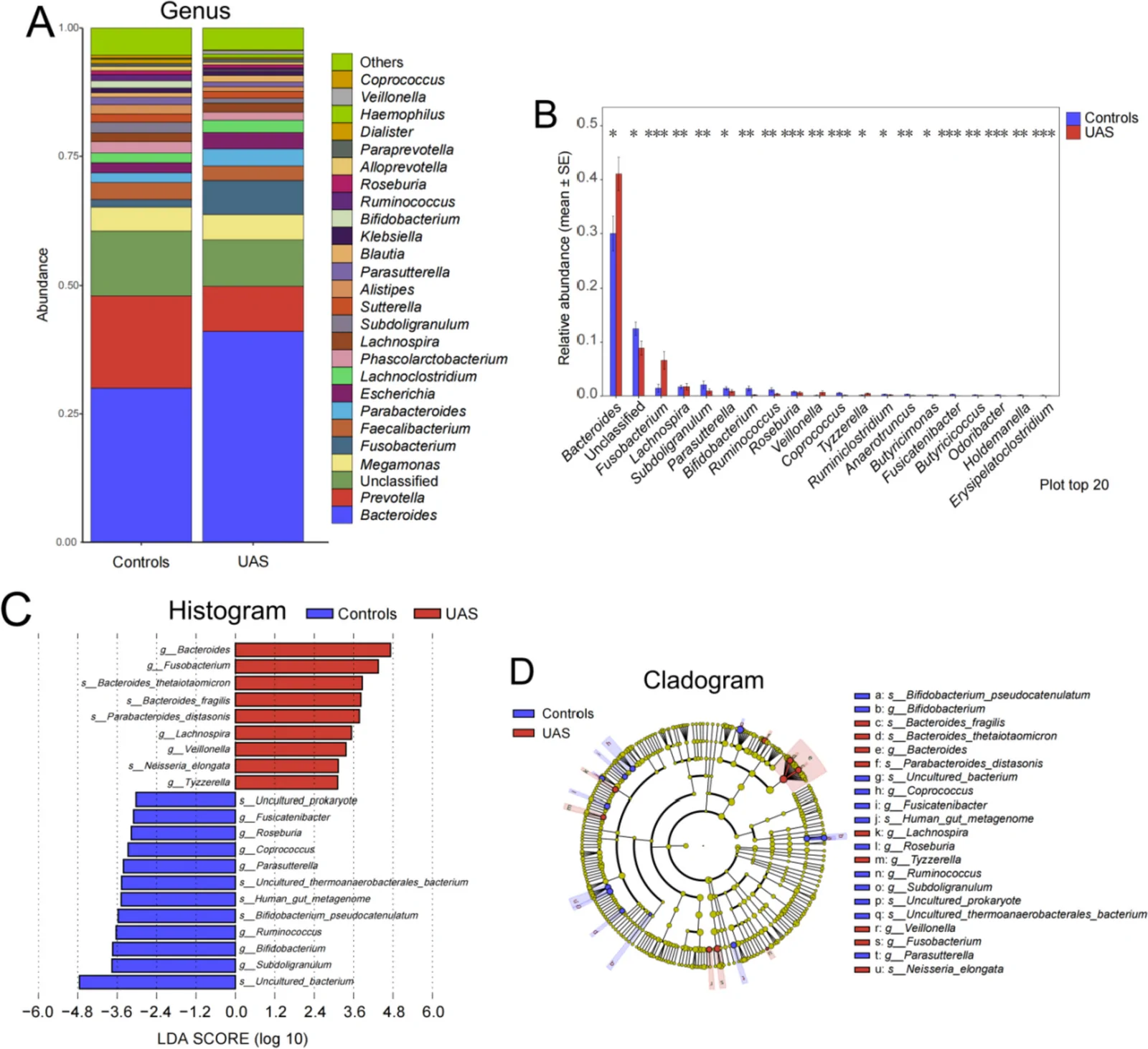
Comparison of relative abundance of flora in samples from UAS and control groups. **A** Results of community analysis at the genus level between UAS and control groups. **B** Histograms depicting variations in the relative abundance of flora at the genus level between UAS and control groups, analyzed using the Kruskal–Wallis test (*P < 0.05, **P < 0.01, ***P < 0.001). **C**, **D** Histograms (Fig. 2C) and cladograms (Fig. 2D) illustrated gut microbiological characteristics specific to UAS patients compared to those observed in the controls at both genus and species levels through linear discriminant analysis of effect sizes (LEfSe) analyses

The taxonomic analysis based on OTUs revealed the species composition from phylum to species level. At the genus level, *Bacteroides*, *Prevotella*, *Megamonas*, *Fusobacterium*, and *Faecalibacterium* were identified as the most abundant genera; however, their relative abundance differed significantly between the two groups. Specifically, *Bacteroides* and *Fusobacterium* were found to be significantly more prevalent while *Subdoligranulum* and *Bifidobacterium* were significantly less abundant in the UAS group compared to NS controls (Fig. 2A–B). The LDA and LEfSe analyses further revealed that, at the genus and species levels, *Bacteroides* including its subspecies, *Fusobacterium* and *Lachnospira*, were found to be the predominant strains in patients with UAS. Conversely, *Subdoligranulum*, *Bifidobacterium* and its subspecies, and *Ruminococcus* emerged as the dominant strains in the control group distinct from those observed in stone patients (Fig. 2C–D).

### Modifications in the clinical features of UAS patients following intervention with PSHC

A total of 17 patients met the indications for PSHC therapy, while 4 patients failed to complete the 3-month therapy cycle. Additionally, one patient received alternative oral medications for a gouty flare-up. Consequently, a total of 12 eligible patients with pure anhydrous uric acid stones remained in the intervention group (Supplemental Fig. S1B). Comparison of clinical features between pre-intervention (preDT group) and post-intervention (DT group) by drug treatment (DT) revealed a significant reduction in serum uric acid levels after dissolution therapy (*P* = 0.025), while no significant difference was observed in serum creatinine levels. The patients demonstrated notable improvement in urinary acidification and a substantial decrease in stone size, indicating successful stone dissolution with oral administration of PSHC (Table 2).

**Table 2:** Clinical features of UAS patients prior to and after treatment with PSHC

Variables	preDT	DT	*P* value
Serum Cr, μmol/L	95.50 (42.25)	93.50 (19.75)	0.817
Serum UA, μmol/L	547.92 ± 90.99	469.17 ± 60.89	**0.021**
Urinary pH	5.00 (0.13)	6.50 ± 0.37	**< 0.001**
Maximum diameter of stone, mm	8.92 ± 2.99	4.33 ± 1.23	**0.001**

Statistical comparisons were performed using both Student’s *t*-test and Mann–Whitney *U* test

*UAS*, uric acid stone; *DT*, drug treatment; *Cr*, creatinine; *UA*, uric acid

Bold values indicate significant differences

### Variation in community diversity, abundance of dominant genera, and SCFAs contents after PSHC intervention

**Fig. 3:**
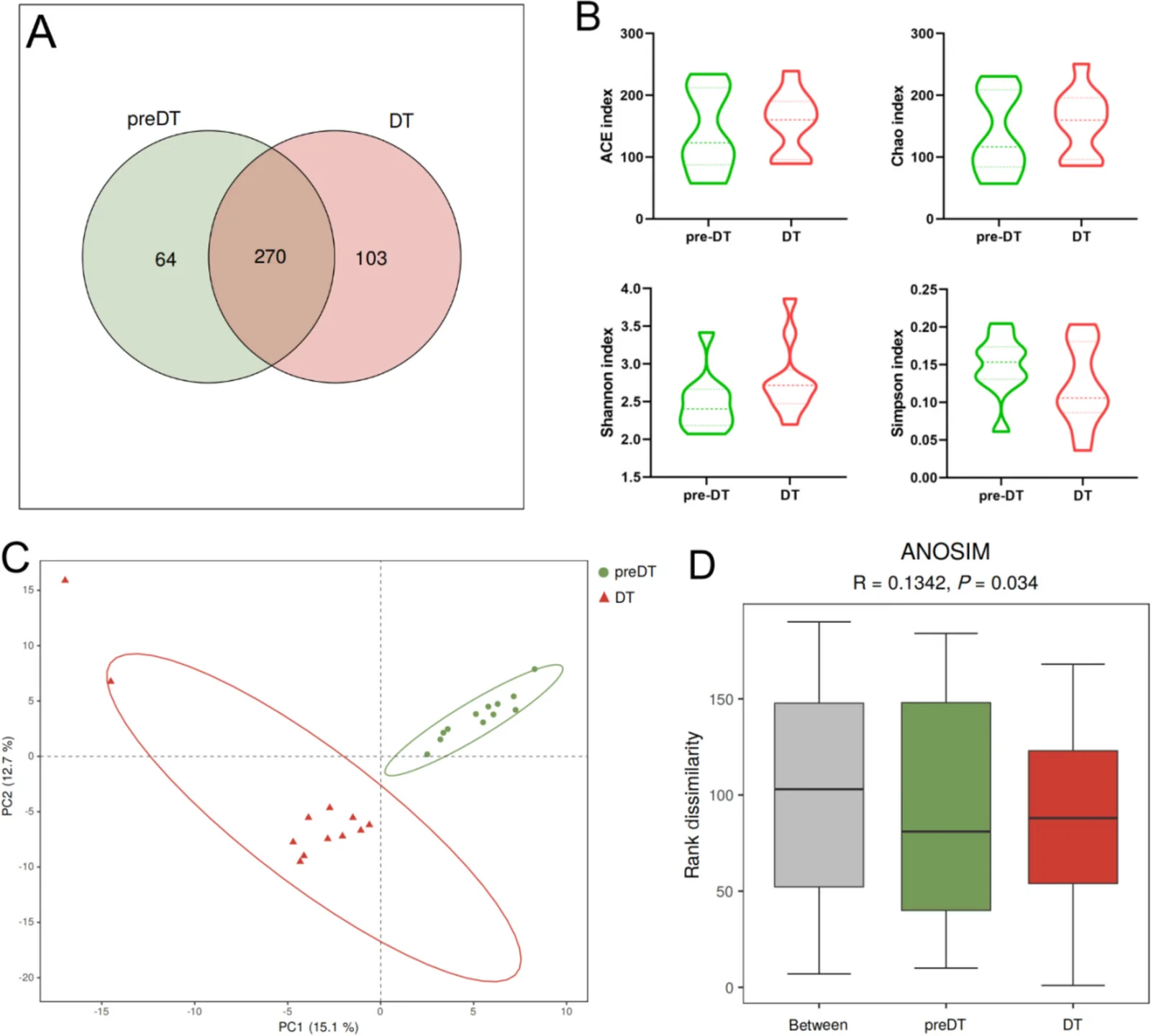
Richness and diversity of gut microbiota in UAS patients before and after the oral dissolution therapy. **A** The Venn diagram depicted the common and unique OTU species between pre-therapy (preDT) and therapy (DT) groups. **B** OTU level-based α-diversity indices between preDT and DT groups determined by Kruskal–Wallis test. **C** In PLS-DA analysis, dots of different colors and shapes represent samples within different groups, and PC1 and PC2 represent possible factors affecting the variation of microbial composition between groups. **D** The abscissa represents all samples (between group) and samples in each group. Anosim analysis revealed that the rank sum of the distance between samples in the between group was higher compared to that in the other two groups, demonstrating that intergroup differences in sample species were greater than intragroup differences and validating the appropriateness of our grouping methodology

Figure 3 exemplifies the diversity and richness variations in the gut microbiota of UAS patients around the time of drug treatment. The Venn diagram in Fig. 3A demonstrated a significant increase in the number of species present in the gut microbiota of UAS patients following drug treatment. The median values of ACE, Chao, and Shannon indices showed an apparent elevation after oral dissolution therapy, but no statistically significant differences were observed (Fig. 3B). Both PLS-DA and Anosim analyses (*R* = 0.134, *P* = 0.034) based on OTU levels revealed substantial disparities in microbiota compositions before and after treatment (Fig. 3C–D).

**Fig. 4:**
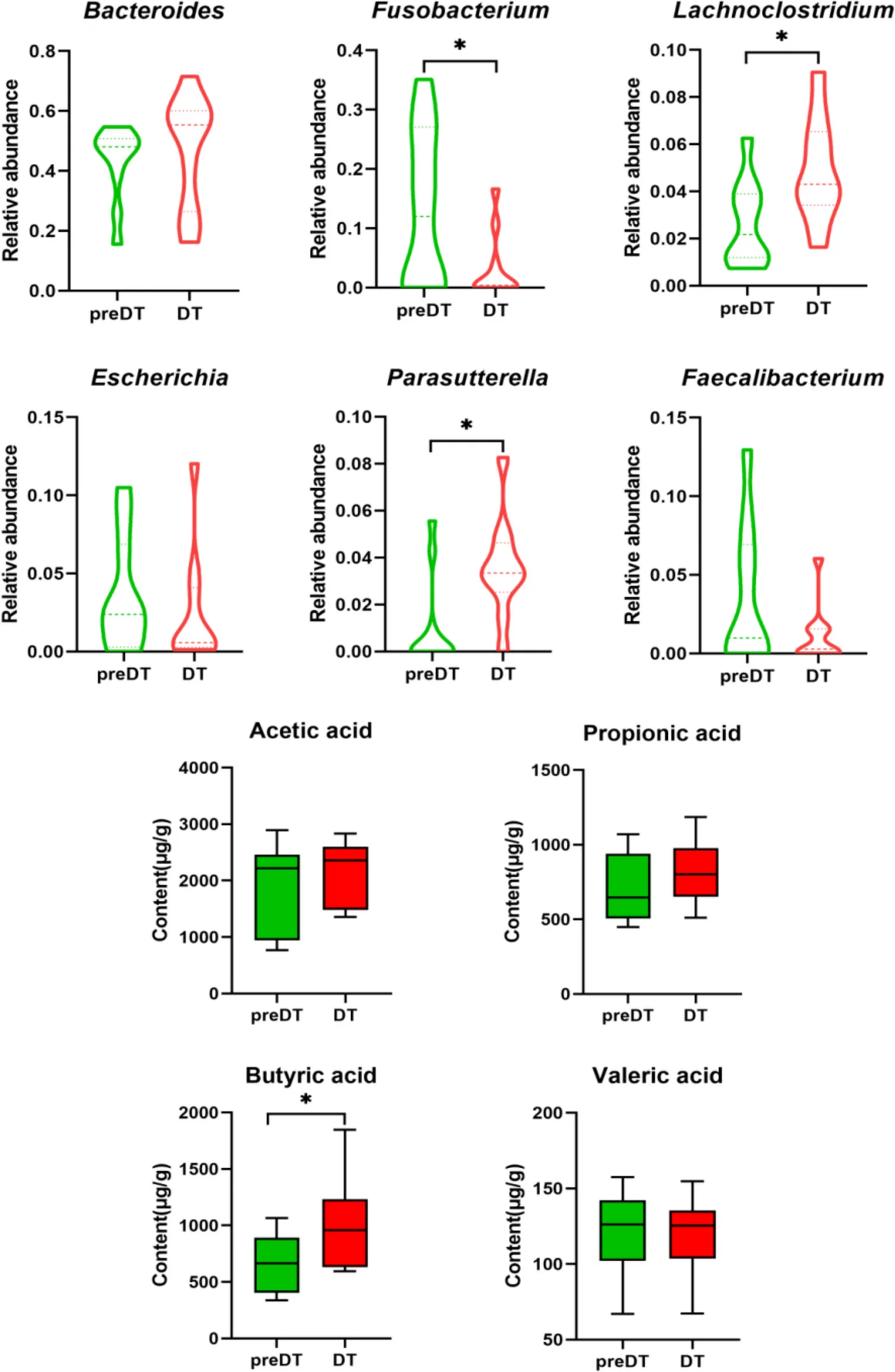
Alterations in the relative abundance of dominant genera and fecal SCFAs contents after PSHC therapy were analyzed by the Kruskal–Wallis test (*P < 0.05)

The OTU-based taxonomic analysis revealed that the genera *Bacteroides*, *Fusobacterium*, *Lachnoclostridium*, *Escherichia*, *Parasutterella*, and *Faecalibacterium* exhibited higher relative abundances at the genus level. However, certain genera displayed significant alterations in abundance following pharmacological treatment. As depicted in Fig. 4, *Fusobacterium*, which was the dominant genus in UAS patients, demonstrated a noteworthy down-regulation after PSHC therapy. Conversely, there was no difference observed in the abundance of *Bacteroides*, another dominant genus. Notably, *Lachnoclostridium* and *Parasutterella* exhibited a substantial upregulation after PSHC therapy. It was worth mentioning that these two upregulated genera were considered core genera involved in gut SCFA synthesis and regulation of signaling pathways (Albuquerque Pereira et al. 2023).

The fecal concentrations of acetic acid, propionic acid, and butyric acid in UAS patients were observed to increase after PSHC therapy; however, a statistically significant difference was only found in the concentration of butyric acid (Fig. 4).

### Prediction of functional and pathway alterations in gut microbiota after PSHC intervention

**Fig. 5:**
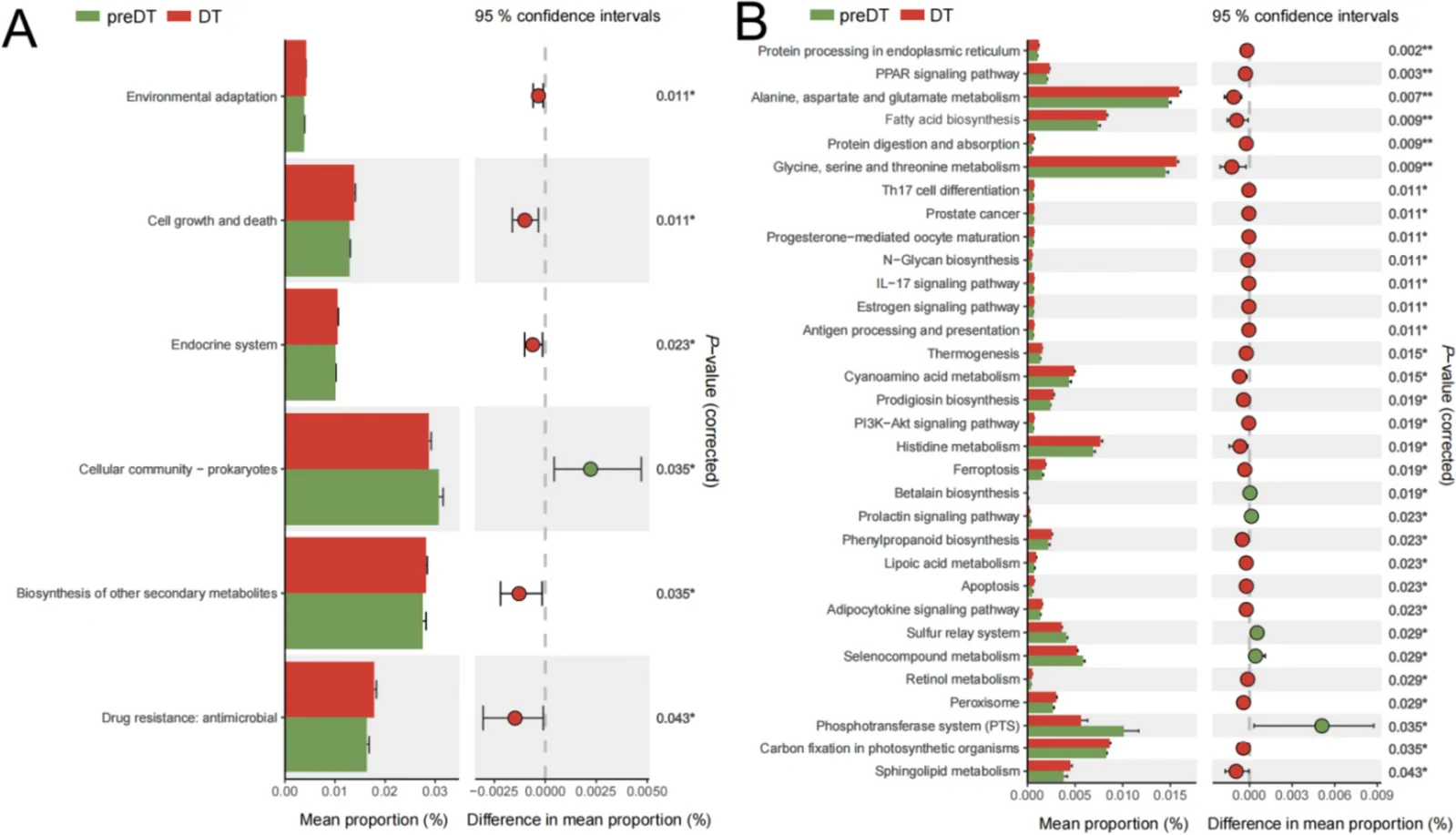
Predicting alteration of gut microbial function after PSHC intervention using PICRUSt. **A** KEGG pathway analysis at the second level revealed upregulation of biosynthetic and metabolic functions in the microbiota after drug intervention. **B** KEGG pathway analysis at the third level demonstrated significant upregulation of fatty acid synthesis and amino acid metabolism in the microbiota following PSHC intervention (analyzed using Kruskal–Wallis test, *P < 0.05, **P < 0.01, ***P < 0.001)

The functional alterations of gut microbiota in UAS patients after PSHC therapy were predicted using PICRUSt. KEGG pathway analysis at the second level indicated a significant upregulation of biosynthetic and metabolic functions in the genera following the intervention (Fig. 5A). Further prediction of metabolism-related pathways at the third level revealed a significant upregulation in biosynthetic functions related to fatty acids, prodigiosin, and phenylpropanoid, as well as metabolic functions associated with amino acids such as alanine, aspartic acid, glutamic acid, glycine, serine, threonine, and histidine within the gut microbiota following the intervention (Fig. 5B).

### Correlation between the abundance of dominant bacterial genera and clinical features, as well as SCFAs contents

**Fig. 6:**
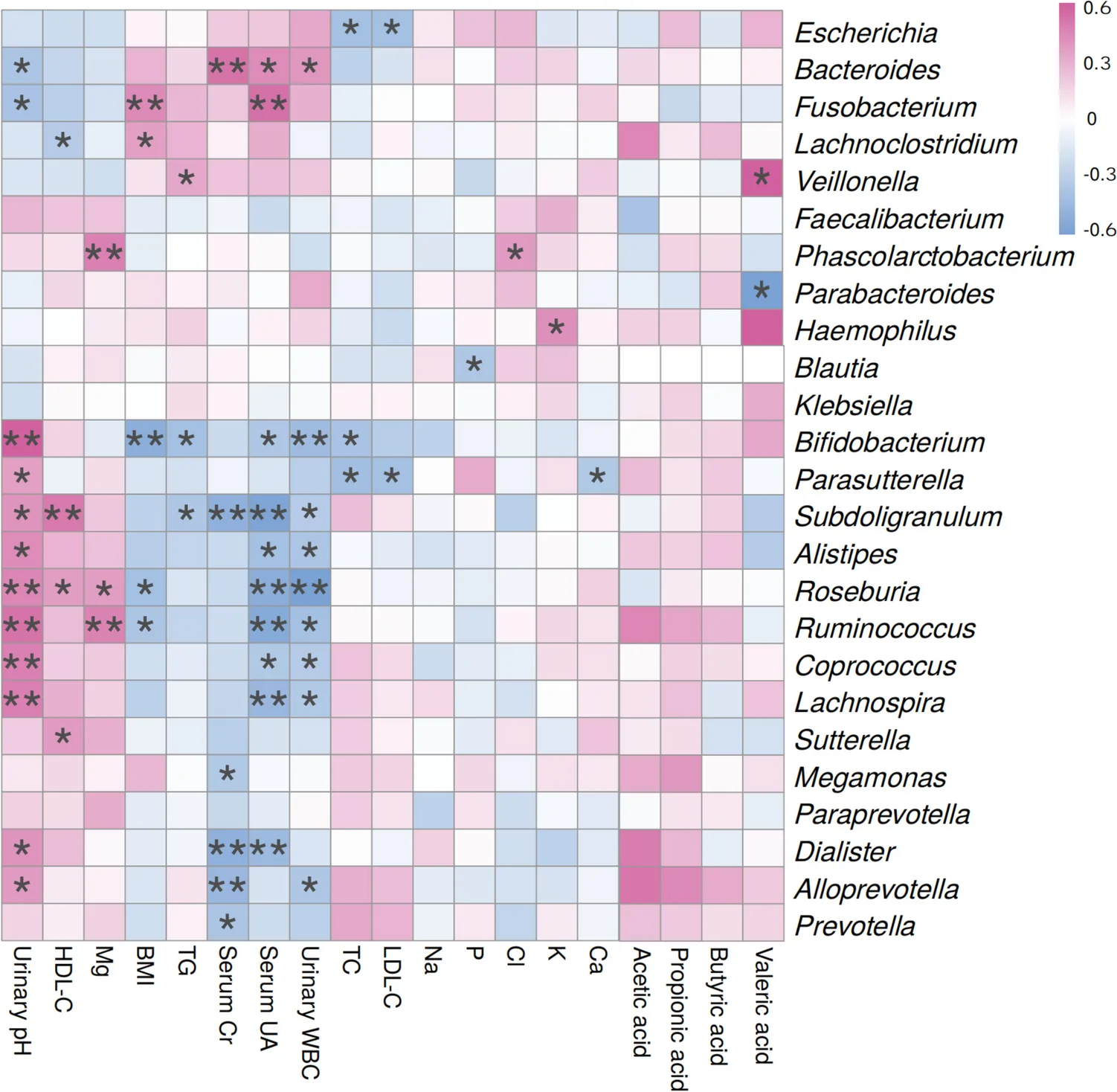
Spearman correlation analysis was performed to investigate the relationship between dominant bacterial genera, clinical features, and SCFAs contents. Positive correlations were represented by red cells while negative correlations were indicated by blue cells. The color depth reflected the degree of relevance, and stars denoted significant correlation (*P < 0.05, **P < 0.01). For abbreviations, please see footnote of Table 1

Based on the sequencing data from all participants, we selected 10 genera out of the top 20 genera with significant differences in relative abundance with LDA values ≥ 3.0. Additionally, we included another 15 dominant gut genera for analysis together. The correlation between the abundance of these 25 genera and various factors such as BMI, serum, and urine biochemical parameters, and SCFAs contents were examined. The abundances of genera such as *Bacteroides* and *Fusobacterium*, which were significantly enriched in the gut of UAS patients, were positively correlated with BMI, serum uric acid, creatinine, triglycerides, and urinary leukocyte count, and negatively correlated with urinary pH levels. In contrast, the abundances of probiotic genera represented by *Bifidobacterium*, *Subdoligranulum*, *Alistipes*, *Roseburia*, and *Ruminococcus* exhibited negative correlations with BMI, serum, and urinary parameters of stone risk factors, and displayed positive correlations with urinary pH levels. It was worth mentioning that these probiotic genera have been shown to directly synthesize SCFAs or modulate signaling pathways involved in SCFAs synthesis. Although not statistically significant, there existed some degree of positive correlation observed between these probiotic genera and fecal acetic, propionic, and butyric acid contents (Fig. 6, Supplemental Table S2).

## Discussion

In our study, patients with UAS presented with metabolic disorders such as hypertension, hyperuricemia, hypertriglyceridemia, and reduced species richness and diversity of gut microbiota. Stone formers had significantly enriched *Bacteroides* and *Fusobacterium* in their gut while probiotic genera like *Subdoligranulum* and *Bifidobacterium* were relatively absent. After 3 months of oral dissolution therapy with PSHC, accompanied by the dissolution of residual kidney stones, serum uric acid levels decreased and urine pH levels increased gradually. The number of species in the gut significantly increased compared to pre-treatment although no differences were observed in species diversity. The abundance of the pathogenic bacterium *Fusobacterium* was downregulated after medication whereas *Lachnoclostridium* and *Parasutterella* involved in SCFA synthesis showed a significant increase in abundance leading to an upregulation of SCFAs contents especially butyric acid in feces post drug therapy. PICRUST functional prediction also verified that fatty acid synthesis capacity of gut microbiota was enhanced significantly after intervention of PSHC. Pathogenic bacteria represented by *Fusobacterium* and *Bacteroides* were positively correlated with clinical stone-forming risk factors while negatively correlated with SCFAs contents which contrasted results for probiotic genera like *Bifidobacterium* and *Subdoligranulum* completely.

Although the prevalence of UAS varies based on population and geography characteristics, numerous epidemiological studies have consistently demonstrated a strong association between metabolic factors and UAS (Liu et al. 2018; Shen et al. 2023; Trinchieri et al. 2020). A large-scale cohort study conducted in China revealed a parallel increase in the incidence of UAS with obesity rates, suggesting that clinical parameters related to metabolic disorders could serve as predictive indicators for their occurrence (Chen et al. 2022). Similarly, a French study observed a higher percentage of UAS among stone patients with a BMI > 30 kg/m^2^ compared to those with a BMI < 25 kg/m^2^, with these stones accounting for 24.1% of all cases in obese individuals (Daudon et al. 2006a). Moreover, diabetic patients face an elevated risk of developing UAS (Abou-Elela 2017; Xiao et al. 2020). Daudon et al. (2006b) found that 35.7% of diabetic patients developed UAS compared to only 11.3% among non-diabetic stone patients. Additionally, dyslipidemia like hypertriglyceridemia and low HDL-cholesterolemia were prevalent among 117 UAS patients included in our single-center retrospective study, further supporting our hypothesis that high uric acid level combined with dyslipidemia may contribute to the pathogenesis through mechanisms involving atherosclerosis and inflammation (Cao et al. 2022).

Previous studies on the pathogenesis of kidney stones have predominantly focused on the physicochemical process of crystallization formation. However, with advancements in high-throughput sequencing technology and metabolomics, increasing attention has been directed toward elucidating the role of gut microbiota in stone formation. In 2016, Stern et al. (2016) reported a 3.4-fold higher abundance of *Bacteroides* in stone patients compared to controls. Another study conducted in India also observed a higher abundance of *Bacteroides* within the gut microbiota of patients suffering from kidney stones, while there was a reduction in *Prevotella*, *Faecalibaderium*, and *Dialister* (Suryavanshi et al. 2016). Tang et al. (2018) conducted a comparative analysis of the gut microbiota in 13 patients with multiple kidney stones and 13 healthy individuals. They identified *Phascolarctobacterium*, *Parasutterella*, *Ruminiclostridium_5*, *Erysipelatoclostridium*, *Fusicatenibacter*, and *Dorea* as specific genera associated with stone formation. In the same year, another study conducted a sequencing analysis of the gut microbiota in 52 patients with recurrent calcium-containing stones. The findings revealed a decreased diversity of gut microbiota in stone-afflicted patients, including the previously mentioned *Prevotella* and *Faecalibaderium*, as well as *Enterobacter* (Ticinesi et al. 2018). Additionally, Zhou et al. (2020) identified a significantly higher incidence of urolithiasis in mice by establishing an animal model of stone formation and feeding them fecal supernatant of stone patients, suggesting that alterations in gut microbiota accelerated renal tubular crystal formation. Another of our recent study found that *Escherichia*, *Fusobacterium*, and *Epulopiscium* were the predominant genera at the genus level in patients with recurrent calcium oxalate stones, while there was a notable decrease in the abundance of *Faecalibacterium*. Moreover, both *Escherichia* and *Fusobacterium* showed significant associations with biochemical indicators and dietary habits among stone-forming patients (Cao et al. 2023). *Oxalobacter formigenes* was also found to play a crucial role by promoting plasma oxalate transport to the intestine and degrading oxalate, thereby reducing urinary oxalate levels and preventing calcium oxalate stone formation (Pebenito et al. 2019). Furthermore, Miller et al. (2019) revealed that *O. formigenes* occupied a central position within an oxalate-metabolizing microbial network involving specialized bacteria as well as parthenogenetic ones such as *Lactobacillus* and *Bifidobacterium* which inhibit stone formation. Nonetheless, the research enthusiasm for *O. formigenes* has been questioned by subsequent studies. In a phase III trial, *O. formigenes* was found to be ineffective in reducing urinary oxalate compared to placebo (Milliner et al. 2018). It has been reported that there was no significant difference in the abundance of *O. formigenes* bacteria between patients with calcium oxalate kidney stones and healthy individuals (Yuan et al. 2022). Unfortunately, most of the aforementioned studies primarily focused on calcium-containing stones, lacking precise classification of stone composition and neglecting investigations into uric acid stones.

In the present study, we observed a reduction in microbiota diversity among UAS patients through 16S rRNA. *Bacteroides* and *Fusobacterium* emerged as the dominant genera in these patients, with the pro-inflammatory effect of *Fusobacterium* potentially contributing to the pathogenesis of UAS. Interestingly, previous studies have reported a significant increase in pro-inflammatory bacteria such as *Megamonas* and *Escherichia* among individuals with renal stones, suggesting that inflammation may play a crucial role in mediating the impact of intestinal flora on stone formation (Tang et al. 2018).

Gut microbiota metabolize dietary components to generate SCFAs, such as acetic acid, propionic acid, butyric acid, and valeric acid (Rios-Covian et al. 2016). These SCFAs play a crucial role in the pathophysiology of blood pressure regulation, acute kidney injury, and chronic kidney disease, which are potentially associated with the development of renal stones (Pluznick 2016). Comparative studies have demonstrated that patients with renal stones exhibit significantly reduced abundance of SCFA-producing microbiota compared to control subjects and also display markedly lower fecal SCFAs contents. This suggests that the absence of SCFA may contribute to renal stone formation through modulation of the gut-kidney axis (Chen et al. 2021a; Hong et al. 2023). Animal experiments further support this notion by revealing that exogenous supplementation with SCFA effectively mitigates calcium oxalate crystal formation in rat kidneys (Liu et al. 2020b). Moreover, our investigation revealed a significant increase in the contents of SCFAs, particularly butyric acid, subsequent to oral dissolution therapy utilizing PSHC among individuals with UAS. Although the precise underlying mechanism remains elusive and necessitates further investigation, these findings underscore the potential efficacy of SCFAs as innovative therapeutic agents for managing kidney stone formation.

Alkalizing the urine is the most crucial approach to promote uric acid stone dissolution. Currently, the primary substances capable of alkalizing urine include citrate, bicarbonate, carbonic anhydrase inhibitors, and certain citrus juice beverages (Cicerello 2018). Oral bicarbonate, which generates carbon dioxide in the presence of gastric acid, is less well-tolerated by patients due to its higher likelihood of causing severe gastrointestinal reactions. Carbonic anhydrase inhibition tends to induce calcium phosphate stone formation as a result of excessive urinary alkalization. Citrate presently serves as a safe and effective agent for urine alkalization (Elbaset et al. 2020; Kamphuis et al. 2019). Compared with sodium citrate and potassium citrate, PSHC exhibits a favorable potassium-to-sodium ratio and is less prone to causing electrolyte imbalances. In this study, patients who completed the 3-month pharmacologic intervention cycle experienced no adverse drug reactions except for mild gastrointestinal responses.

Salem et al. (2019) conducted a prospective study on 139 patients with stone sizes ranging from 5 to 30 mm and stone computed tomography (CT) scan values < 600 Hounsfield unit (HU) who were treated with PSHC oral dissolution, which resulted in an overall effective rate of 64.8%. Mokhless et al. (2009) reported the results of treating 24 cases of radiolucent renal stones in children with oral PSHC stone dissolution combined with ESWL, achieving a 100% stone clearance rate. Elderwy et al. (2014) reported a stone-free rate of 73% at 1–3 months for radiolucent renal stones treated with PSHC, which is comparable to the outcomes achieved through ESWL. In our study, after completing 3 months of oral dissolution therapy, patients showed an approximately 50% reduction in maximum diameter of stones and significant improvement in urine pH above 6.0. Meanwhile, these patients exhibited moderate reductions in serum uric acid levels even in the absence of uric acid-lowering medications. The reason for this, in addition to the patients’ dietary management during treatment, warrants further investigation as to whether PSHC increases uric acid excretion.

After 3 months of PSHC intervention, our study revealed a significant decrease in the abundance of *Fusobacterium*, which is typically enriched in the gut of UAS patients. However, there was no notable change observed in the abundance of another dominant genus, *Bacteroides*. Meanwhile, the core intestinal genera such as *Lachnoclostridium* and *Parasutterella* were upregulated. *Lachnoclostridium* is a Gram-positive genus that specializes in anaerobic fermentation to produce SCFAs like butyric and acetic acids (Dou et al. 2023). These substances are crucial for renal protection due to their anti-inflammatory, anti-atherosclerotic, and antioxidant effects. The development of various metabolic diseases including obesity, hypertension, and diabetes have been closely linked to *Lachnoclostridium* (Zang et al. 2023; Zhao et al. 2023), which can be increased by GLP-1 receptor agonists used to treat diabetes (Tsai et al. 2021). Our study demonstrated that PSHC can also increase the abundance of *Lachnoclostridium* for the first time. *Parasutterella*, another core intestinal bacterial genus, exerts an impact on the host flora and metabolism by inducing changes in metabolites such as aromatic amino acids, bilirubin, purine, and bile acid derivatives within the mouse intestine. High abundance of *Parasutterella* has been associated with activation of the SCFA synthesis pathway in humans (Ju et al. 2019).

*Fusobacterium* represents Gram-negative anaerobic bacteria that can interact with other microorganisms, leading to opportunistic pathogenesis. With the advancement of microbiome technology, numerous diseases have been closely associated with *Fusobacterium* infection, including atherosclerotic cardiovascular disease (ASCVD) (Zhou et al. 2022), inflammatory bowel disease (IBD) (Liu et al. 2020a), oral squamous cell carcinoma (He et al. 2023), gastrointestinal cancers (Liu et al. 2019), and even kidney stones (Cao et al. 2023). *Fusobacterium* is a potent cytokine stimulating factor that causes persistent localized infections and induces upregulation of the inflammatory cascade response. Moreover, it promotes macrophage M1-type polarization by up-regulating Toll-like receptors (TLRs) expression, enhances CD4 + T-cell proliferation and differentiation into Th1 and Th17 cells, and facilitates the progression of several systemic diseases (Nejman et al. 2020). Several studies have demonstrated that SCFAs contents in particular butyrate which has therapeutic effects on stones are significantly negatively correlated with the abundance of *Fusobacterium* in the intestine (Calatayud et al. 2022; Du et al. 2022; Kong et al. 2023).

Moreover, the Spearman’s correlation heatmap revealed a positive correlation between stone-forming bacteria, such as *Bacteroides* and *Fusobacterium*, and stone risk factors including BMI, serum creatinine, serum uric acid, serum triglycerides, and urine leukocytes levels. Conversely, these bacteria were negatively correlated with protective factors like serum HDL-C and urine pH levels. In contrast to the stone-causing bacteria mentioned above, intestinal probiotic genera such as *Bifidobacterium* and *Subdoligranulum* exhibited negative associations with the aforementioned risk factors but positive relationships with protective factors such as serum HDL-C and serum magnesium levels. Unfortunately, there were no significant upregulation in the abundance of *Bifidobacterium* and *Subdoligranulum* following PSHC intervention due to both small sample size limitations and uncertainties regarding drug action mechanisms.

Hyperuricemia exacerbates the burden of uric acid excretion in urine, leading to impaired ammonia secretion and subsequent acidification of urine, which promotes crystal deposition through inflammatory injury and oxidative stress in renal tubular epithelial cells. *Fusobacterium* induces a pro-inflammatory micro-environment that inhibits SCFA synthesis, aggravating the inflammatory response and immune stress in renal tubular epithelial cells. This results in impaired ammonia secretion and urate transport. However, after PSHC intervention, the abundance of *Fusobacterium* is significantly suppressed, leading to upregulation of SCFAs such as butyric acid. The activation of specific signaling pathways by SCFA mitigates inflammatory injury in renal tubular epithelial cells, restores ammonia secretion function, and potentially accelerates urate transport and reabsorption while reducing urinary acidity and levels of urinary urate. The modulation of SCFAs contents by *Fusobacterium difficile* has an impact on inflammation and oxidative stress in renal tubular epithelial cells. However, the precise mechanism through which inflammatory factors contribute to urine acidification and subsequent stone formation remains unclear. Could targeting the metabolic pathways associated with gut microbiota-SCFA enhance drug therapy efficacy? Further studies are required to validate these scientific inquiries.

There are several limitations to this study. Firstly, the sample size of included studies was insufficient due to the rarity of UAS patients and strict criteria for flora analysis. Specifically, in the PSHC intervention study, only 12 out of 17 patients completed the 3-month treatment cycle, and further validation is required by expanding the sample size and prolonging the treatment cycle in future studies. Secondly, owing to financial limitations, the contents of SCFAs were only measured in patients receiving pharmacological intervention, whereas it was not evaluated among other participants. Thirdly, additional validation is required to establish a causal relationship between PSHC intervention and alterations in gut microbiota as well as SCFA by constructing animal models of urolithiasis.

In conclusion, we investigated the diversity and composition of gut microbiota in UAS patients to gain insights into their characteristics. By implementing PSHC intervention in UAS patients, alternations in microflora diversity, abundance of core bacteria, and levels of fecal SCFAs were observed after treatment. Moreover, our investigation anticipated modifications in the metabolic function of microbiota post-treatment and established the association between bacterial genera and clinical parameters. It is imperative to conduct large-scale, long-term, and multi-center cohort studies, as well as establish animal models for the purpose of validating the causal relationship between drug effects and gut microbiota as well as metabolites. This study offers novel perspectives and therapeutic targets for preventing and treating UAS. To our knowledge, this is the first prospective cohort study analyzing gut microbiota characteristics in PSHC-treated patients with UAS.

## Supplementary Information

Below is the link to the electronic supplementary material.

Supplementary file1 (PDF 328 KB)

## Data Availability

The raw data of 16S rRNA gene sequencing were uploaded as a BioProject to the NCBI database (PRJNA1001580) (https://www.ncbi. nlm.nih.gov/Traces/study/?acc = SRP453091&o = acc_s%3Aa).

## References

[CR1] Abou-Elela A (2017) Epidemiology, pathophysiology, and management of uric acid urolithiasis: a narrative review. J Adv Res 8(5):513–527. 10.1016/j.jare.2017.04.005 28748117 10.1016/j.jare.2017.04.005 PMC5512151

[CR2] Abufaraj M, Al Karmi J, Yang L (2022) Prevalence and trends of urolithiasis among adults. Curr Opin Urol 32(4):425–432. 10.1097/MOU.0000000000000994 35703251 10.1097/MOU.0000000000000994

[CR3] Albuquerque Pereira MF, Morais de Avila LG, Avila Alpino GC, Dos Santos Cruz BC, Almeida LF, Macedo Simoes J, Ladeira Bernardes A, Xisto Campos I, de Oliveira Barros Ribon A, de Oliveira Mendes TA, Gouveia Peluzio MDC (2023) Milk kefir alters fecal microbiota impacting gut and brain health in mice. Appl Microbiol Biotechnol 107(16):5161–5178. 10.1007/s00253-023-12630-0 37389589 10.1007/s00253-023-12630-0

[CR4] Bamberger JN, Rosen DC, Khusid JA, Kaplan-Marans E, Gallante B, Kapoor A, Paranjpe I, Atashsokhan DJ, Atallah WM, Gupta M (2021) The impact of metabolic syndrome components on urinary parameters and risk of stone formation. World J Urol 39(12):4483–4490. 10.1007/s00345-021-03790-7 34264364 10.1007/s00345-021-03790-7

[CR5] Calatayud M, Xiong C, Selma-Royo M, van de Wiele T (2022) Arsenolipids reduce butyrate levels and influence human gut microbiota in a donor-dependent way. Ecotoxicol Environ Saf 246:114175. 10.1016/j.ecoenv.2022.114175 36252516 10.1016/j.ecoenv.2022.114175

[CR6] Cao C, Fan B, Zhu J, Zhu N, Cao JY, Yang DR (2022) Association of gut microbiota and biochemical features in a Chinese population with renal uric acid stone. Front Pharmacol 13:888883. 10.3389/fphar.2022.888883 35662733 10.3389/fphar.2022.888883 PMC9160931

[CR7] Cao C, Jin X, Ding Q, Zhu J, Yang D, Fan B (2023) The altered composition of gut microbiota and biochemical features as well as dietary patterns in a southern Chinese population with recurrent renal calcium oxalate stones. Urolithiasis 51(1):95. 10.1007/s00240-023-01467-x 37458823 10.1007/s00240-023-01467-x

[CR8] Chen F, Bao X, Liu S, Ye K, Xiang S, Yu L, Xu Q, Zhang Y, Wang X, Zhu X, Ying J, Shen Y, Ji W, Si S (2021a) Gut microbiota affect the formation of calcium oxalate renal calculi caused by high daily tea consumption. Appl Microbiol Biotechnol 105(2):789–802. 10.1007/s00253-020-11086-w 33404827 10.1007/s00253-020-11086-w

[CR9] Chen Y, Zhou J, Wang L (2021b) Role and mechanism of gut microbiota in human disease. Front Cell Infect Microbiol 11:625913. 10.3389/fcimb.2021.625913 33816335 10.3389/fcimb.2021.625913 PMC8010197

[CR10] Chen HW, Chen YC, Lee JT, Yang FM, Kao CY, Chou YH, Chu TY, Juan YS, Wu WJ (2022) Prediction of the uric acid component in nephrolithiasis using simple clinical information about metabolic disorder and obesity: a machine learning-based model. Nutrients 14(9). 10.3390/nu14091829 10.3390/nu14091829 PMC9103478 35565794

[CR11] Cicerello E (2018) Uric acid nephrolithiasis: an update. Urologia 85(3):93–98. 10.1177/0391560318766823 29687761 10.1177/0391560318766823

[CR12] Daudon M, Lacour B, Jungers P (2006) Influence of body size on urinary stone composition in men and women. Urol Res 34(3):193–199. 10.1007/s00240-006-0042-8 16474948 10.1007/s00240-006-0042-8

[CR13] Daudon M, Traxer O, Conort P, Lacour B, Jungers P (2006b) Type 2 diabetes increases the risk for uric acid stones. J Am Soc Nephrol 17(7):2026–2033. 10.1681/ASN.2006030262 16775030 10.1681/ASN.2006030262

[CR14] Dou L, Liu C, Chen X, Yang Z, Hu G, Zhang M, Sun L, Su L, Zhao L, Jin Y (2023) Supplemental *Clostridium butyricum* modulates skeletal muscle development and meat quality by shaping the gut microbiota of lambs. Meat Sci 204:109235. 10.1016/j.meatsci.2023.109235 37301103 10.1016/j.meatsci.2023.109235

[CR15] Du Y, Li X, An Y, Song Y, Lu Y (2022) Association of gut microbiota with sort-chain fatty acids and inflammatory cytokines in diabetic patients with cognitive impairment: a cross-sectional, non-controlled study. Front Nutr 9:930626. 10.3389/fnut.2022.930626 35938126 10.3389/fnut.2022.930626 PMC9355148

[CR16] Elbaset MA, Hashem A, Eraky A, Badawy MA, El-Assmy A, Sheir KZ, Shokeir AA (2020) Optimal non-invasive treatment of 1-2.5 cm radiolucent renal stones: oral dissolution therapy, shock wave lithotripsy or combined treatment-a randomized controlled trial. World J Urol 38(1):207–212. 10.1007/s00345-019-02746-2 30944968 10.1007/s00345-019-02746-2

[CR17] Elderwy AA, Kurkar A, Hussein A, Abozeid H, Hammodda HM, Ibraheim AF (2014) Dissolution therapy versus shock wave lithotripsy for radiolucent renal stones in children: a prospective study. J Urol 191(5 Suppl):1491–1495. 10.1016/j.juro.2013.10.060 24679880 10.1016/j.juro.2013.10.060

[CR18] Elsawy AA, Elshal AM, El-Nahas AR, Elbaset MA, Farag H, Shokeir AA (2019) Can we predict the outcome of oral dissolution therapy for radiolucent renal calculi? A Prospect Study J Urol 201(2):350–357. 10.1016/j.juro.2018.09.027 10.1016/j.juro.2018.09.027 30218763

[CR19] He X, Ma X, Meng Z, Han Z, Chen W (2023) Localization of *Fusobacterium* nucleatum in oral squamous cell carcinoma and its possible directly interacting protein molecules: a case series. Histol Histopathol 38(8):929–939. 10.14670/HH-18-560 36478348 10.14670/HH-18-560

[CR20] Hong SY, Xia QD, Yang YY, Li C, Zhang JQ, Xu JZ, Qin BL, Xun Y, Wang SG (2023) The role of microbiome: a novel insight into urolithiasis. Crit Rev Microbiol 49(2):177–196. 10.1080/1040841X.2022.2045899 35776498 10.1080/1040841X.2022.2045899

[CR21] Ju T, Kong JY, Stothard P, Willing BP (2019) Defining the role of *Parasutterella*, a previously uncharacterized member of the core gut microbiota. ISME J 13(6):1520–1534. 10.1038/s41396-019-0364-5 30742017 10.1038/s41396-019-0364-5 PMC6776049

[CR22] Kamphuis GM, Wouter van Hattum J, de Bie P, Somani BK (2019) Method of alkalization and monitoring of urinary pH for prevention of recurrent uric acid urolithiasis: a systematic review. Transl Androl Urol 8(Suppl 4):S448–S456. 10.21037/tau.2019.05.01 31656751 10.21037/tau.2019.05.01 PMC6790419

[CR23] Kong C, Liang L, Liu G, Du L, Yang Y, Liu J, Shi D, Li X, Ma Y (2023) Integrated metagenomic and metabolomic analysis reveals distinct gut-microbiome-derived phenotypes in early-onset colorectal cancer. Gut 72(6):1129–1142. 10.1136/gutjnl-2022-327156 35953094 10.1136/gutjnl-2022-327156

[CR24] Liu Y, Chen Y, Liao B, Luo D, Wang K, Li H, Zeng G (2018) Epidemiology of urolithiasis in Asia. Asian J Urol 5(4):205–214. 10.1016/j.ajur.2018.08.007 30364478 10.1016/j.ajur.2018.08.007 PMC6197415

[CR25] Liu Y, Baba Y, Ishimoto T, Iwatsuki M, Hiyoshi Y, Miyamoto Y, Yoshida N, Wu R, Baba H (2019) Progress in characterizing the linkage between *Fusobacterium nucleatum* and gastrointestinal cancer. J Gastroenterol 54(1):33–41. 10.1007/s00535-018-1512-9 30244399 10.1007/s00535-018-1512-9

[CR26] Liu H, Hong XL, Sun TT, Huang XW, Wang JL, Xiong H (2020a) *Fusobacterium nucleatum* exacerbates colitis by damaging epithelial barriers and inducing aberrant inflammation. J Dig Dis 21(7):385–398. 10.1111/1751-2980.12909 32441482 10.1111/1751-2980.12909

[CR27] Liu Y, Jin X, Hong HG, Xiang L, Jiang Q, Ma Y, Chen Z, Cheng L, Jian Z, Wei Z, Ai J, Qi S, Sun Q, Li H, Li Y, Wang K (2020b) The relationship between gut microbiota and short chain fatty acids in the renal calcium oxalate stones disease. FASEB J 34(8):11200–11214. 10.1096/fj.202000786R 32645241 10.1096/fj.202000786R

[CR28] Ma Q, Fang L, Su R, Ma L, Xie G, Cheng Y (2018a) Uric acid stones, clinical manifestations and therapeutic considerations. Postgrad Med J 94(1114):458–462. 10.1136/postgradmedj-2017-135332 30002092 10.1136/postgradmedj-2017-135332

[CR29] Ma RH, Luo XB, Li Q, Zhong HQ (2018b) Systemic analysis of urinary stones from the northern, eastern, central, southern and southwest China by a multi-center study. BMC Urol 18(1):114. 10.1186/s12894-018-0428-2 30545321 10.1186/s12894-018-0428-2 PMC6293513

[CR30] Miller AW, Choy D, Penniston KL, Lange D (2019) Inhibition of urinary stone disease by a multi-species bacterial network ensures healthy oxalate homeostasis. Kidney Int 96(1):180–188. 10.1016/j.kint.2019.02.012 31130222 10.1016/j.kint.2019.02.012 PMC6826259

[CR31] Milliner D, Hoppe B, Groothoff J (2018) A randomised phase II/III study to evaluate the efficacy and safety of orally administered *Oxalobacter formigenes* to treat primary hyperoxaluria. Urolithiasis 46(4):313–323. 10.1007/s00240-017-0998-6 28718073 10.1007/s00240-017-0998-6 PMC6061479

[CR32] Mokhless IA, Sakr MA, Abdeldaeim HM, Hashad MM (2009) Radiolucent renal stones in children: combined use of shock wave lithotripsy and dissolution therapy. Urology 73(4):772–775. 10.1016/j.urology.2008.10.066 19193412 10.1016/j.urology.2008.10.066

[CR33] Nejman D, Livyatan I, Fuks G, Gavert N, Zwang Y, Geller LT, Rotter-Maskowitz A, Weiser R, Mallel G, Gigi E, Meltser A, Douglas GM, Kamer I, Gopalakrishnan V, Dadosh T, Levin-Zaidman S, Avnet S, Atlan T, Cooper ZA, Arora R, Cogdill AP, Khan MAW, Ologun G, Bussi Y, Weinberger A, Lotan-Pompan M, Golani O, Perry G, Rokah M, Bahar-Shany K, Rozeman EA, Blank CU, Ronai A, Shaoul R, Amit A, Dorfman T, Kremer R, Cohen ZR, Harnof S, Siegal T, Yehuda-Shnaidman E, Gal-Yam EN, Shapira H, Baldini N, Langille MGI, Ben-Nun A, Kaufman B, Nissan A, Golan T, Dadiani M, Levanon K, Bar J, Yust-Katz S, Barshack I, Peeper DS, Raz DJ, Segal E, Wargo JA, Sandbank J, Shental N, Straussman R (2020) The human tumor microbiome is composed of tumor type-specific intracellular bacteria. Science 368(6494):973–980. 10.1126/science.aay9189 32467386 10.1126/science.aay9189 PMC7757858

[CR34] Nevo A, Levi O, Sidi A, Tsivian A, Baniel J, Margel D, Lifshitz D (2020) Patients treated for uric acid stones reoccur more often and within a shorter interval compared to patients treated for calcium stones. Can Urol Assoc J 14(11):E555–E559. 10.5489/cuaj.6259 32520701 10.5489/cuaj.6259 PMC7673824

[CR35] Pebenito AM, Liu M, Nazzal L, Blaser MJ (2019) Development of a humanized murine model for the study of *Oxalobacter formigenes* intestinal colonization. J Infect Dis 220(11):1848–1858. 10.1093/infdis/jiz370 31328778 10.1093/infdis/jiz370 PMC6804336

[CR36] Pluznick JL (2016) Gut microbiota in renal physiology: focus on short-chain fatty acids and their receptors. Kidney Int 90(6):1191–1198. 10.1016/j.kint.2016.06.033 27575555 10.1016/j.kint.2016.06.033 PMC5123942

[CR37] Ramos GK, Goldfarb DS (2022) Update on uric acid and the kidney. Curr Rheumatol Rep 24(5):132–138. 10.1007/s11926-022-01069-3 35420373 10.1007/s11926-022-01069-3

[CR38] Rios-Covian D, Ruas-Madiedo P, Margolles A, Gueimonde M, de Los Reyes-Gavilan CG, Salazar N (2016) Intestinal short chain fatty acids and their link with diet and human health. Front Microbiol 7:185. 10.3389/fmicb.2016.00185 26925050 10.3389/fmicb.2016.00185 PMC4756104

[CR39] Salem SM, Sultan MF, Badawy A (2019) Oral dissolution therapy for renal radiolucent stones, outcome, and factors affecting response: a prospective study. Urol Ann 11(4):369–373. 10.4103/UA.UA_20_19 31649455 10.4103/UA.UA_20_19 PMC6798288

[CR40] Scales CD Jr, Smith AC, Hanley JM, Saigal CS, Urologic Diseases in America P (2012) Prevalence of kidney stones in the United States. Eur Urol 62(1):160–165. 10.1016/j.eururo.2012.03.052 22498635 10.1016/j.eururo.2012.03.052 PMC3362665

[CR41] Shen X, Pan Q, Huang Y, You J, Chen Y, Ding X (2023) Metabolic syndrome predicts uric acid stones in the upper urinary tract: development and validation of a nomogram model. Arch Esp Urol 76(4):255–263. 10.56434/j.arch.esp.urol.20237604.29 37455524 10.56434/j.arch.esp.urol.20237604.29

[CR42] Sorokin I, Mamoulakis C, Miyazawa K, Rodgers A, Talati J, Lotan Y (2017) Epidemiology of stone disease across the world. World J Urol 35(9):1301–1320. 10.1007/s00345-017-2008-6 28213860 10.1007/s00345-017-2008-6

[CR43] Stern JM, Moazami S, Qiu Y, Kurland I, Chen Z, Agalliu I, Burk R, Davies KP (2016) Evidence for a distinct gut microbiome in kidney stone formers compared to non-stone formers. Urolithiasis 44(5):399–407. 10.1007/s00240-016-0882-9 27115405 10.1007/s00240-016-0882-9 PMC8887828

[CR44] Suryavanshi MV, Bhute SS, Jadhav SD, Bhatia MS, Gune RP, Shouche YS (2016) Hyperoxaluria leads to dysbiosis and drives selective enrichment of oxalate metabolizing bacterial species in recurrent kidney stone endures. Sci Rep 6:34712. 10.1038/srep34712 27708409 10.1038/srep34712 PMC5052600

[CR45] Tang RQ, Jiang YH, Tan AH, Ye J, Xian XY, Xie YL, Wang QY, Yao ZT, Mo ZN (2018) 16S rRNA gene sequencing reveals altered composition of gut microbiota in individuals with kidney stones. Urolithiasis 46(6):503–514. 10.1007/s00240-018-1037-y 29353409 10.1007/s00240-018-1037-y

[CR46] Ticinesi A, Milani C, Guerra A, Allegri F, Lauretani F, Nouvenne A, Mancabelli L, Lugli GA, Turroni F, Duranti S, Mangifesta M, Viappiani A, Ferrario C, Dodi R, Dall’Asta M, Del Rio D, Ventura M, Meschi T (2018) Understanding the gut-kidney axis in nephrolithiasis: an analysis of the gut microbiota composition and functionality of stone formers. Gut 67(12):2097–2106. 10.1136/gutjnl-2017-315734 29705728 10.1136/gutjnl-2017-315734

[CR47] Trinchieri A, Croppi E, Simonelli G, Sciorio C, Montanari E (2020) Anthropometric variables, physical activity and dietary intakes of patients with uric acid nephrolithiasis. Urolithiasis 48(2):123–129. 10.1007/s00240-019-01138-w 31037403 10.1007/s00240-019-01138-w

[CR48] Tsai CY, Lu HC, Chou YH, Liu PY, Chen HY, Huang MC, Lin CH, Tsai CN (2021) Gut microbial signatures for glycemic responses of GLP-1 receptor agonists in type 2 diabetic patients: a pilot study. Front Endocrinol (lausanne) 12:814770. 10.3389/fendo.2021.814770 35095773 10.3389/fendo.2021.814770 PMC8793908

[CR49] Turk C, Petrik A, Sarica K, Seitz C, Skolarikos A, Straub M, Knoll T (2016) EAU guidelines on diagnosis and conservative management of urolithiasis. Eur Urol 69(3):468–474. 10.1016/j.eururo.2015.07.040 26318710 10.1016/j.eururo.2015.07.040

[CR50] Xiao Y, Wei L, Xiong X, Yang Y, Li L, Yang M, Deng F, Sun L (2020) Sex differences in kidney stone disease in Chinese patients with type 2 diabetes mellitus. Kidney Dis (basel) 6(3):195–203. 10.1159/000506053 32523961 10.1159/000506053 PMC7265720

[CR51] Yasui T, Iguchi M, Suzuki S, Kohri K (2008) Prevalence and epidemiological characteristics of urolithiasis in Japan: national trends between 1965 and 2005. Urology 71(2):209–213. 10.1016/j.urology.2007.09.034 18308085 10.1016/j.urology.2007.09.034

[CR52] Ye Z, Zeng G, Yang H, Li J, Tang K, Wang G, Wang S, Yu Y, Wang Y, Zhang T, Long Y, Li W, Wang C, Wang W, Gao S, Shan Y, Huang X, Bai Z, Lin X, Cheng Y, Wang Q, Xu Z, Xie L, Yuan J, Ren S, Fan Y, Pan T, Wang J, Li X, Chen X, Gu X, Sun Z, Xiao K, Jia J, Zhang Q, Wang G, Sun T, Li X, Xu C, Xu C, Shi G, He J, Song L, Sun G, Wang D, Liu Y, Wang C, Han Y, Liang P, Wang Z, He W, Chen Z, Xing J, Xu H (2020) The status and characteristics of urinary stone composition in China. BJU Int 125(6):801–809. 10.1111/bju.14765 30958622 10.1111/bju.14765

[CR53] Yuan C, Jin X, He Y, Liu Y, Xiang L, Wang K (2022) Association of dietary patterns with gut microbiota in kidney stone and non-kidney stone individuals. Urolithiasis 50(4):389–399. 10.1007/s00240-022-01325-2 35460343 10.1007/s00240-022-01325-2

[CR54] Zang L, Baharlooeian M, Terasawa M, Shimada Y, Nishimura N (2023) Beneficial effects of seaweed-derived components on metabolic syndrome via gut microbiota modulation. Front Nutr 10:1173225. 10.3389/fnut.2023.1173225 37396125 10.3389/fnut.2023.1173225 PMC10311452

[CR55] Zhao R, Li N, Liu W, Liu Q, Zhang L, Peng X, Zhao R, Hu H (2023) Low glycemic index potato biscuits alleviate physio-histological damage and gut dysbiosis in rats with type-2 diabetes mellitus induced by high-sugar and high-fat diet and streptozotocin. J Nutr Biochem 119:109401. 10.1016/j.jnutbio.2023.109401 37276891 10.1016/j.jnutbio.2023.109401

[CR56] Zhou C, Li K, Zhao L, Li W, Guo Z, Xu J, Qi X, Yuan H (2020) The relationship between urinary stones and gut microbiomeby 16S sequencing. Biomed Res Int 2020:1582187. 10.1155/2020/1582187 33083452 10.1155/2020/1582187 PMC7556066

[CR57] Zhou J, Liu L, Wu P, Zhao L, Wu Y (2022) *Fusobacterium nucleatum* accelerates atherosclerosis via macrophage-driven aberrant proinflammatory response and lipid metabolism. Front Microbiol 13:798685. 10.3389/fmicb.2022.798685 35359716 10.3389/fmicb.2022.798685 PMC8963492

