# Supplementary material for: Potassium sodium hydrogen citrate intervention on gut microbiota and clinical features in uric acid stone patients
Source: Appl Microbiol Biotechnol. 2024 Jan 6;108:51. doi: 10.1007/s00253-023-12953-y (PMC10771603; doi:10.1007/s00253-023-12953-y)
Supplement: Supplementary file 1 [file 253_2023_12953_MOESM1_ESM.pdf]

Applied Microbiology and Biotechnology

Supplemental Material

Potassium sodium hydrogen citrate intervention on gut microbiota and clinical features in uric acid stone patients

Cheng Cao[1†] Feng Li[1†] Qi Ding[1] Xiaohua Jin[1] Wenjian Tu[1] Hailiang Zhu[1] Mubin Sun[1] Jin Zhu[2] Dongrong Yang[2] Bo Fan[1]*

[1]Department of Urology, The Changshu Hospital Affiliated to Soochow University (Changshu No.1 People's Hospital), Changshu, China;

[2]Department of Urology, The Second Affiliated Hospital of Soochow University, Suzhou, China;

*Corresponding author. E-mail address: fanbosuda@163.com (B. Fan).

[†]These authors have contributed equally to this work.

Supplemental Table S1. Mean dietary nutrient intake over a span of three consecutive days

Intake of nutrients	Contents（/day）
Total energy (kcal)	2115.6±85.9
Protein (g)	88.5±0.5
Fat (g)	55.0±0.9
Carbohydrate (g)	312.3±18.7
Total dietary fiber (g)	25.9±1.9
Vitamin A (μg)	758.4±43.3
Vitamin B1 (mg)	1.4±0.1
Vitamin B2 (mg)	1.2±0.1
Vitamin C (mg)	96.0±3.2
Vitamin E (mg)	14.3±0.9
Carotene (μg)	2575.3±74.7
Ca (mg)	963.3±82.1
K (mg)	1768.6±132.5
Na (mg)	1366.9±110.3
Mg (mg)	326.0±16.5
Fe (mg)	19.8±1.3
Zn (mg)	12.5±0.7
Cu (mg)	0.7±0.1
P (mg)	835.5±18.7
Se (μg)	51.7±2.8

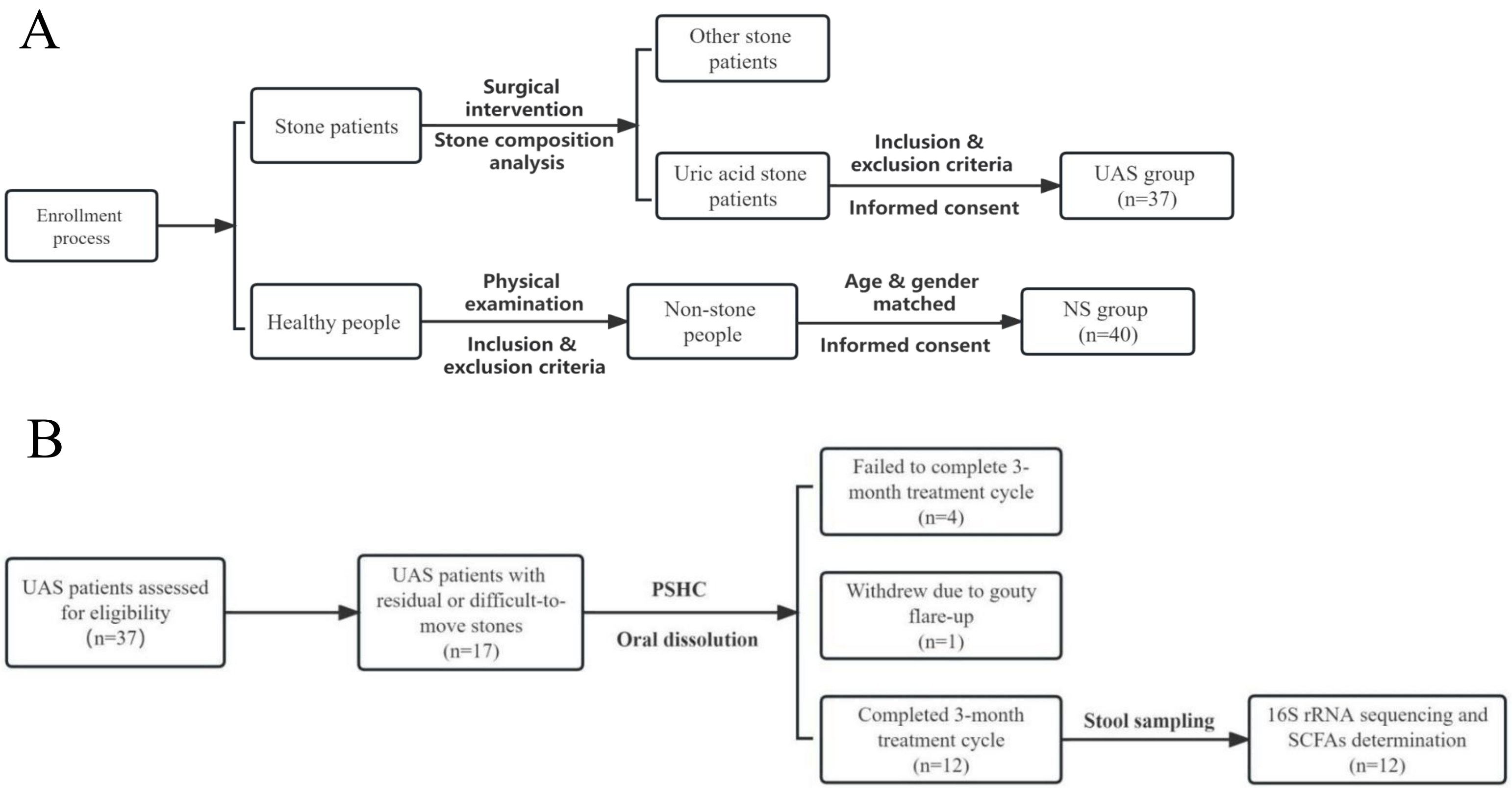

Supplemental Fig. S1 Flowchart of the study recruitment. A. Enrollment protocol for patients diagnosed with uric acid stones. B. Enrollment process during the PSHC intervention phase

Supplemental Table S2. Correlation analysis between dominant bacterial genera, clinical features and SCFAs contents

Variables	*Alistipes*		*Alloprevotella*		*Bacteroides*		*Bifidobacterium*		*Blautia*	
	r	***P***	***r***	***P***	***r***	***P***	***r***	***P***	***r***	***P***
BMI	-0.223	0.051	-0.111	0.337	0.193	0.093	-0.352	**0.002**	-0.023	0.843
TG (mmol/L)	-0.183	0.110	0.065	0.572	0.101	0.381	-0.277	**0.015**	0.051	0.661
TC (mmol/L)	-0.033	0.773	0.200	0.081	-0.198	0.084	-0.265	**0.020**	-0.120	0.300
HDL-C (mmol/L)	0.187	0.104	0.049	0.675	-0.181	0.116	0.108	0.351	0.031	0.789
LDL-C (mmol/L)	-0.088	0.447	0.164	0.155	-0.119	0.303	-0.222	0.052	-0.123	0.285
K (mmol/L)	0.005	0.963	-0.120	0.299	0.110	0.342	-0.116	0.316	0.159	0.166
Na (mmol/L)	-0.126	0.273	-0.091	0.432	0.075	0.515	-0.193	0.093	0.076	0.511
Cl (mmol/L)	-0.038	0.743	-0.129	0.263	0.127	0.271	-0.056	0.629	0.124	0.283
Ca (mmol/L)	-0.021	0.854	-0.045	0.700	-0.035	0.762	-0.049	0.675	0.019	0.867
P (mmol/L)	-0.099	0.391	-0.103	0.372	-0.012	0.917	-0.048	0.677	-0.245	**0.032**
Mg (mmol/L)	0.157	0.173	0.034	0.770	-0.125	0.280	-0.086	0.455	0.071	0.537
Cr (µmol/L)	-0.163	0.157	-0.312	**0.006**	0.354	**0.002**	-0.166	0.149	0.023	0.842
UA (µmol/L)	-0.278	**0.014**	-0.119	0.302	0.288	**0.011**	-0.248	**0.029**	-0.002	0.986
Urinary pH	0.285	**0.012**	0.257	**0.024**	-0.249	**0.029**	0.406	**0.000**	-0.118	0.305
Urinary WBC (/µL)	-0.251	**0.028**	-0.244	**0.032**	0.266	**0.019**	-0.295	**0.009**	-0.018	0.879
Acetic acid (µg/g)	0.122	0.608	0.379	0.100	0.071	0.767	-0.047	0.845	0.012	0.960
Propionic acid (µg/g)	0.089	0.710	0.309	0.184	0.021	0.930	0.056	0.816	0.095	0.691
Butyric acid (µg/g)	0.128	0.591	0.211	0.373	-0.048	0.840	0.080	0.738	-0.069	0.772
Valeric acid (µg/g)	-0.333	0.152	0.110	0.643	-0.009	0.970	0.221	0.348	0.205	0.387

Variables	*Coprococcus*		*Dialister*		*Escherichia*		*Faecalibacterium*		*Fusobacterium*	
	r	***P***	***r***	***P***	***r***	***P***	***r***	***P***	***r***	***P***
BMI	-0.148	0.198	-0.087	0.453	0.026	0.821	-0.091	0.432	0.297	**0.009**
TG (mmol/L)	-0.089	0.440	-0.041	0.726	0.006	0.957	-0.081	0.484	0.182	0.114
TC (mmol/L)	0.145	0.209	0.000	0.998	-0.275	**0.015**	-0.047	0.688	-0.073	0.529
HDL-C (mmol/L)	0.124	0.281	0.166	0.149	-0.151	0.189	0.152	0.186	-0.211	0.065
LDL-C (mmol/L)	0.088	0.448	-0.045	0.700	-0.276	**0.015**	-0.116	0.315	0.003	0.976
K (mmol/L)	0.080	0.487	-0.195	0.090	-0.107	0.353	0.195	0.089	0.015	0.896
Na (mmol/L)	-0.154	0.180	0.112	0.332	0.058	0.618	-0.071	0.538	-0.010	0.928
Cl (mmol/L)	-0.073	0.526	-0.146	0.205	0.184	0.110	0.126	0.273	0.067	0.560
Ca (mmol/L)	0.071	0.541	-0.096	0.406	-0.085	0.462	0.043	0.712	0.117	0.312
P (mmol/L)	-0.086	0.455	0.009	0.938	0.155	0.177	-0.022	0.851	0.092	0.428
Mg (mmol/L)	0.131	0.256	0.015	0.894	-0.149	0.195	0.151	0.191	-0.140	0.223
Cr (μmol/L)	-0.154	0.180	-0.343	**0.002**	0.134	0.244	-0.043	0.712	0.144	0.212
UA (μmol/L)	-0.236	**0.039**	-0.302	**0.008**	0.136	0.238	-0.162	0.160	0.363	**0.001**
Urinary pH	0.322	**0.004**	0.268	**0.018**	-0.142	0.219	0.183	0.112	-0.258	**0.024**
Urinary WBC (/μL)	-0.229	**0.045**	-0.117	0.311	0.218	0.057	-0.072	0.532	0.199	0.082
Acetic acid (μg/g)	-0.035	0.855	0.355	0.124	-0.192	0.416	-0.371	0.108	-0.030	0.900
Propionic acid (μg/g)	0.094	0.694	0.174	0.462	0.167	0.482	-0.043	0.857	-0.265	0.259
Butyric acid (μg/g)	0.048	0.842	-0.152	0.521	-0.176	0.458	-0.034	0.887	-0.158	0.516
Valeric acid (μg/g)	-0.013	0.956	-0.025	0.916	0.183	0.439	-0.093	0.697	-0.173	0.466

Variables	*Haemophilus*		*Klebsiella*		*Lachnoclostridium*		*Lachnospira*		*Megamonas*	
	r	***P***	***r***	***P***	***r***	***P***	***r***	***P***	***r***	***P***
BMI	0.067	0.564	-0.009	0.935	0.242	**0.034**	-0.203	0.077	0.180	0.117
TG (mmol/L)	0.112	0.331	0.079	0.493	0.185	0.107	-0.057	0.624	-0.015	0.896
TC (mmol/L)	-0.091	0.429	0.021	0.853	-0.116	0.315	0.122	0.291	0.132	0.253
HDL-C (mmol/L)	-0.004	0.971	0.008	0.947	-0.227	**0.048**	0.194	0.091	0.098	0.397
LDL-C (mmol/L)	-0.160	0.164	0.025	0.832	0.029	0.802	0.061	0.598	0.108	0.351
K (mmol/L)	0.277	**0.015**	0.097	0.399	-0.023	0.842	0.000	0.998	0.074	0.524
Na (mmol/L)	-0.035	0.762	0.010	0.930	-0.047	0.683	0.093	0.421	-0.006	0.962
Cl (mmol/L)	0.006	0.960	0.035	0.759	0.045	0.697	-0.139	0.230	-0.051	0.657
Ca (mmol/L)	0.035	0.762	-0.068	0.554	-0.013	0.908	0.059	0.612	0.061	0.599
P (mmol/L)	0.027	0.819	-0.027	0.818	-0.033	0.773	-0.078	0.499	0.101	0.382
Mg (mmol/L)	0.046	0.688	0.004	0.975	-0.072	0.531	0.114	0.322	0.032	0.783
Cr (μmol/L)	-0.033	0.774	0.022	0.851	0.032	0.780	-0.179	0.120	-0.232	**0.042**
UA (μmol/L)	0.032	0.780	-0.057	0.623	0.205	0.074	-0.308	**0.006**	-0.031	0.788
Urinary pH	-0.050	0.667	-0.147	0.202	-0.116	0.315	0.317	**0.005**	0.059	0.607
Urinary WBC (/μL)	0.110	0.343	-0.027	0.814	-0.051	0.661	-0.240	**0.036**	-0.026	0.821
Acetic acid (μg/g)	0.090	0.707	0.008	0.842	0.319	0.171	0.036	0.879	0.214	0.364
Propionic acid (μg/g)	0.091	0.702	0.012	0.891	0.023	0.925	0.142	0.549	0.269	0.252
Butyric acid (μg/g)	-0.086	0.718	0.020	0.719	0.165	0.486	-0.183	0.439	-0.037	0.879
Valeric acid (μg/g)	0.442	0.051	0.000	0.995	-0.042	0.860	0.130	0.584	0.039	0.871

Variables	*Parabacteroides*		*Paraprevotella*		*Parasutterella*		*Phascolarctobacterium*		*Prevotella*	
	r	***P***	***r***	***P***	***r***	***P***	***r***	***P***	***r***	***P***
BMI	0.074	0.524	-0.086	0.457	-0.110	0.342	-0.083	0.475	-0.147	0.202
TG (mmol/L)	0.037	0.746	-0.039	0.738	-0.139	0.229	-0.008	0.942	0.032	0.779
TC (mmol/L)	-0.047	0.684	0.123	0.287	-0.285	**0.012**	-0.021	0.859	0.221	0.053
HDL-C (mmol/L)	0.096	0.407	0.080	0.490	-0.057	0.620	0.068	0.554	0.045	0.698
LDL-C (mmol/L)	-0.146	0.204	0.068	0.559	-0.275	**0.015**	-0.074	0.522	0.188	0.102
K (mmol/L)	-0.021	0.857	-0.029	0.805	0.076	0.513	0.097	0.402	0.047	0.683
Na (mmol/L)	0.031	0.791	-0.198	0.084	0.004	0.975	-0.108	0.350	-0.054	0.643
Cl (mmol/L)	0.168	0.145	-0.157	0.173	-0.046	0.691	0.259	**0.023**	-0.186	0.106
Ca (mmol/L)	-0.046	0.693	-0.097	0.402	-0.240	**0.036**	0.031	0.788	-0.038	0.744
P (mmol/L)	0.056	0.628	0.064	0.580	0.213	0.063	-0.077	0.504	0.056	0.627
Mg (mmol/L)	0.041	0.722	0.207	0.071	0.084	0.469	0.317	**0.005**	0.112	0.331
Cr (μmol/L)	0.052	0.656	-0.170	0.139	-0.052	0.653	0.026	0.820	-0.249	**0.029**
UA (μmol/L)	-0.018	0.876	-0.090	0.437	-0.116	0.317	0.007	0.951	-0.151	0.191
Urinary pH	-0.063	0.585	0.114	0.323	0.235	**0.040**	0.095	0.412	0.110	0.343
Urinary WBC (/μL)	0.217	0.058	0.011	0.923	-0.212	0.065	-0.146	0.206	-0.200	0.081
Acetic acid (μg/g)	-0.132	0.578	-0.076	0.751	0.162	0.496	-0.220	0.351	0.146	0.539
Propionic acid (μg/g)	-0.191	0.420	0.036	0.879	0.035	0.884	0.082	0.730	0.110	0.645
Butyric acid (μg/g)	0.108	0.650	0.020	0.932	0.115	0.630	0.056	0.813	0.048	0.840
Valeric acid (μg/g)	-0.558	**0.011**	-0.148	0.533	-0.092	0.699	-0.214	0.365	0.078	0.743

Variables	*Roseburia*		*Ruminococcus*		*Subdoligranulum*		*Sutterella*		*Veillonella*	
	r	***P***	***r***	***P***	***r***	***P***	***r***	***P***	***r***	***P***
BMI	-0.277	**0.015**	-0.256	**0.025**	-0.196	0.088	-0.053	0.648	0.070	0.548
TG (mmol/L)	-0.114	0.323	-0.186	0.106	-0.245	**0.032**	-0.074	0.520	0.226	**0.048**
TC (mmol/L)	0.009	0.938	0.006	0.959	0.167	0.147	0.125	0.279	0.014	0.904
HDL-C (mmol/L)	0.248	**0.030**	0.164	0.155	0.335	**0.003**	0.257	**0.024**	-0.119	0.302
LDL-C (mmol/L)	-0.046	0.691	0.006	0.957	0.070	0.542	0.033	0.774	-0.019	0.868
K (mmol/L)	0.012	0.919	0.096	0.407	-0.004	0.971	-0.095	0.410	0.021	0.854
Na (mmol/L)	-0.042	0.714	-0.022	0.851	-0.043	0.709	-0.022	0.847	0.009	0.935
Cl (mmol/L)	-0.041	0.723	0.028	0.808	-0.215	0.060	0.074	0.523	-0.037	0.748
Ca (mmol/L)	0.118	0.305	0.067	0.563	0.030	0.794	0.146	0.205	0.128	0.269
P (mmol/L)	-0.078	0.498	-0.139	0.228	0.011	0.927	-0.092	0.428	-0.168	0.145
Mg (mmol/L)	0.248	**0.030**	0.315	**0.005**	0.142	0.218	0.193	0.092	-0.147	0.201
Cr (μmol/L)	-0.161	0.163	-0.154	0.181	-0.342	**0.002**	-0.215	0.061	0.147	0.204
UA (μmol/L)	-0.356	**0.001**	-0.365	**0.001**	-0.390	**0.000**	-0.124	0.283	0.163	0.157
Urinary pH	0.305	**0.007**	0.352	**0.002**	0.274	**0.016**	0.126	0.275	-0.115	0.318
Urinary WBC (/μL)	-0.412	**0.000**	-0.274	**0.016**	-0.228	**0.046**	-0.127	0.272	0.143	0.214
Acetic acid (μg/g)	-0.191	0.420	0.323	0.165	-0.118	0.620	0.008	0.974	-0.118	0.620
Propionic acid (μg/g)	0.009	0.970	0.232	0.325	0.008	0.972	0.052	0.827	-0.083	0.729
Butyric acid (μg/g)	-0.038	0.874	0.174	0.464	0.094	0.694	-0.211	0.371	-0.119	0.618
Valeric acid (μg/g)	-0.070	0.770	-0.143	0.548	-0.331	0.154	-0.214	0.366	0.450	**0.047**
